# Gm527 deficiency in dentate gyrus improves memory through upregulating dopamine D1 receptor pathway

**DOI:** 10.1111/cns.14259

**Published:** 2023-05-29

**Authors:** Jie Jia, Hualing Peng, Rui Tian, Hong Zhou, Hua Zheng, Bo Liu, Yisheng Lu

**Affiliations:** ^1^ Department of Physiology, School of Basic Medicine Tongji Medical College, Huazhong University of Science and Technology Wuhan China; ^2^ Department of Anesthesiology, Hubei Key Laboratory of Geriatric Anesthesia and Perioperative Brain Health, and Wuhan Clinical Research Center for Geriatric Anesthesia Tongji Hospital, Tongji Medical College, Huazhong University of Science and Technology Wuhan China; ^3^ Department of Otorhinolaryngology, Union Hospital Tongji Medical College, Huazhong University of Science and Technology Wuhan China; ^4^ Institute of Brain Research, Collaborative Innovation Center for Brain Science Huazhong University of Science and Technology Wuhan China

**Keywords:** adult neurogenesis, D1 receptor, Gm527, long‐term memory, NMDA, working memory

## Abstract

**Aims:**

Dopamine D1 receptor (D1R) hypofunction is associated with negative and cognitive symptoms in schizophrenia; therefore, the mechanism of D1R function modulation needs further investigation. Gm527 is the rodent homologous of the schizophrenia‐related gene C14orf28, encoding a predicated D1R‐interacting protein. However, the role of Gm527‐D1R interaction in schizophrenia needs to be clarified.

**Methods:**

Gm527‐floxed mice were generated and crossed with D1‐Cre mice (D1:Gm527−/−) to knockout Gm527 in D1R‐positive neurons. Then behavioral tests were performed to explore the schizophrenia‐related phenotypes. Immunofluorescence, fluorescence in situ hybridization, electrophysiological recording, quantitative real‐time PCR, and western blotting were conducted to investigate the mechanisms.

**Results:**

Working memory, long‐term memories, and adult neurogenesis in the DG were enhanced in D1:Gm527−/− mice. LTP was also increased in the DG in D1:Gm527−/− mice, resulting from the Gm527 knockout‐induced D1R expression enhancement on the plasma membrane and subsequently cAMP signaling and NMDA receptor pathways activation. The requirement of Gm527 knockout in the DG was confirmed by reversing Gm527 expression or knockdown Gm527 in the DG D1R‐positive neurons through AAV‐CAG‐FLEX‐Gm527‐GFP or AAV‐CMV‐FLEX‐EGFP‐Gm527‐RNAi injection.

**Conclusions:**

The DG Gm527 knockout induces D1R hyperfunction in improving schizophrenia cognitive symptoms.

## Graphical abstract

Knockout Gm527 activates D1R downstream pathways including cAMP signaling and NMDA receptors, promotes synaptic plasticity in the dentate gyrus, resulting in improvement of adult neurogenesis, working memory, and long‐term memory.

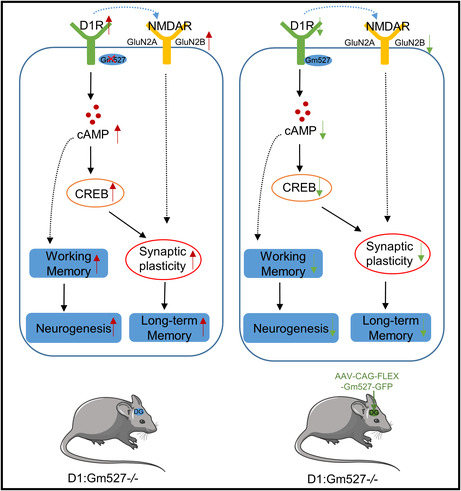

## 1 INTRODUCTION

Dopaminergic neurotransmission in the brain has major roles in cognition, affection, and locomotion; pathology in the dopamine system underlies several psychiatric diseases, including schizophrenia.^1^, ^2^ Dopamine receptors are G‐protein‐coupled receptors, classified into Gαs‐coupled D1‐ (D1 and D5) and Gαi/o‐coupled D2‐ (D2, D3, and D4) family, promoting or inhibiting the production of cAMP respectively.^3^, ^4^ The “dopamine hypothesis” of schizophrenia suggests that the positive symptoms (hallucinations and delusions) of schizophrenia result from the hyperactive dopamine transmission in the mesolimbic areas, mainly mediated by D2 receptors; and the negative symptoms (anhedonia and lack of motivation) result from the hypoactive dopamine transmission in the prefrontal cortex (PFC), mainly mediated by D1 receptors.^5^, ^6^, ^7^ For cognitive symptoms, the hippocampus and PFC are involved in working memory and episodic memory deficits^8^, ^9^; however, how the dopamine transmission involves in these two brain regions is still largely unknown, especially in the hippocampus.

The dentate gyrus (DG) is the input region of the hippocampus, essential for the representations of sensory information^10^, ^11^ and memory formation.^12^ Adult neurogenesis in the subgranular zone of the DG plays an essential role in cognitive functions,^13^ and its impairment is involved in neuropsychiatric disorders, such as bipolar disorder and schizophrenia.^14^, ^15^ Dopamine guides the memory encoding^16^ in the DG, which might be due to the modulation of D1R in the plasticity of glutamatergic neuron synapses from perforant path to DG granular layer.^17^ Besides regulating cAMP production, the D1R interacts with NMDA receptors, required for memory formation and synaptic plasticity.^18^ However, it is still controversial that the D1R is a schizophrenia‐related gene,^19^ whether malfunction of D1R in the DG can induce schizophrenia‐related phenotypes needs further investigation.

Dopamine receptors interact with dopamine receptor‐interacting proteins (DRIPs),^20^, ^21^ which can assist dopamine receptors maturation, control the intracellular signal transduction in space and time in different cell types.^22^ Recently, the exon sequencing study of schizophrenia patients and their parents found that C14orf28 (chromosome 14 open reading frame 28) might be a schizophrenia‐related gene,^23^ and its expression is significantly increased in schizophrenia patients.^24^ Its homologous gene Gm527 in mice encoded protein shares 96% amino acid sequence homology with C14orf28, indicating a highly conserved function. Gm527 directly binds to D1R, tested by yeast two‐hybrid experiments,^24^ whether Gm527 is a D1R DRIP protein needs to be confirmed, and its mechanisms in the D1R regulation and schizophrenia pathology remain unknown.

To clarify the role of Gm527 in D1R‐positive neurons, we generated Gm527‐floxed mice and crossed with D1‐Cre mice to specifically knockout Gm527 in the D1R‐positive neurons. Interestingly, adult neurogenesis, spatial working memory, and long‐term memories were improved. To investigate the mechanisms, we found that Gm527 knockout in D1R‐positive neurons increased D1R expression on the plasma membrane, which might subsequently enhance NMDA receptor and cAMP signaling pathway and then facilitate the LTP in the DG, leading to memory and neurogenesis enhancement. These results suggest that Gm527 is also a DRIP of D1R, inhibits D1R expression on the plasma membrane, and is involved in schizophrenia in a “gain‐of‐function” manner.

## 2 MATERIALS AND METHODS

### 2.1 Animals

**FIGURE 1:**
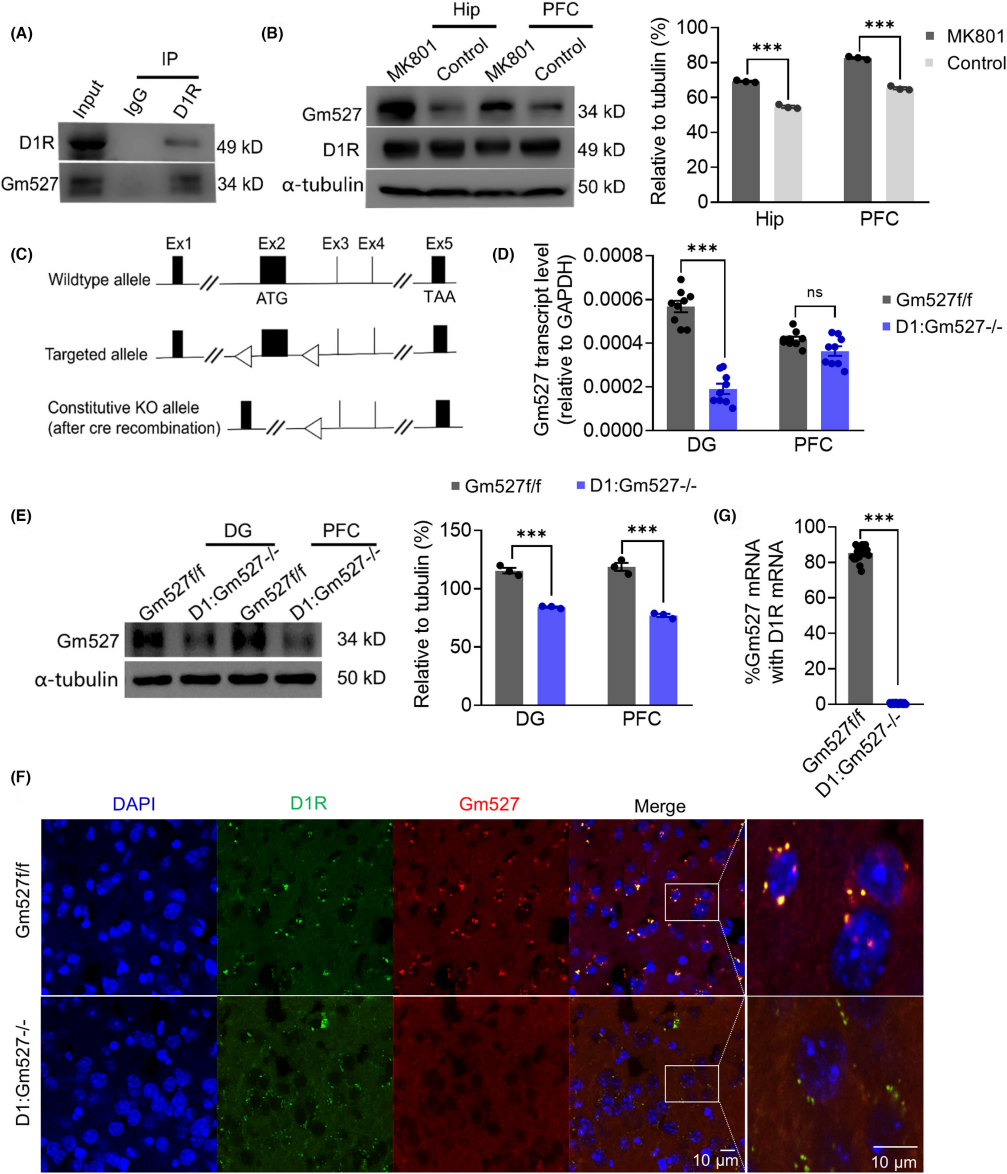
The expression of Gm527 decreases in the DG of D1:Gm527−/− mice. (A) Gm527 interacted with D1R tested by immunoprecipitation. (B) Left, the protein levels of Gm527 in the hippocampal and PFC were increased in MK801‐injected mice. Right, quantification of Gm527 levels (*n* = 3–4 mice/group, two‐way ANOVA, *F* (1, 8) = 755.4, *p* < 0.0001). (C) Generation of the Gm527‐floxed mice. Gm527 gene has five exons with the start codon ATG in exon 2 and the TAA stop codon in exon 5. Two loxP sites were inserted flanking exon 2. After Cre combination, the loxP region was deleted and the function of Gm527 gene was lost. (D) Gm527 transcript level was decreased in the DG of D1:Gm527−/− mice, but not in the PFC, revealed by qRT‐PCR analysis (*n* = 3 mice/group in triplicate, two‐way ANOVA, *F* (1, 32) = 100.7, *p* < 0.0001). (E) Left, Gm527 protein levels were decreased in the DG, PFC of D1:Gm527−/− mice, revealed by western blot analysis. Right, quantification of Gm527 levels (*n* = 3–4 mice/group, two‐way ANOVA, *F* (1, 8) = 262.9, *p* < 0.0001). (F) Representative images showing the D1R and Gm527 mRNA in the DG of the D1:Gm527−/− mice, tested by FISH. D1R and Gm527 mRNAs were detected by hybridization chain reaction (HCR) that simultaneously used different probe–hairpin pairs: Alexa488‐conjugated hairpin DNA for D1R and Alexa546‐conjugated hairpin DNA for Gm527. (G) The expression of Gm527 mRNA in the DG was decreased in D1:Gm527−/− mice (five mice per group and five sections per mouse, Mann–Whitney test, *p* < 0.0001).

C57BL/6J and D1‐Cre mice (stock No: 037156) were purchased from Jackson Laboratory. The loxP‐flanked (floxed)‐Gm527 mouse is customized by Cyagen Biotechnology Co., Ltd., which inserts two loxP sites flanking exon 2 (Figure 1c). D1‐Cre mice were crossed with Gm527‐floxed mice to generate D1‐Cre:Gm527‐floxed f/f (D1:Gm527−/−) mice. The Institutional Animal Ethics Committee of Huazhong University of Science and Technology approved all the experimental procedures with mice.

Less than five mice were housed per cage at 22–24°C and 55%–80% humidity, on a schedule of 12:12 h light/dark cycle, with water and food available ad libitum. All mice were backcrossed with C57BL/6J mice for more than 10 generations.

### 2.2 Slice electrophysiology

After mice were anesthetized with 1% pentobarbital sodium (35 mg/kg, i.p.), the brains were quickly removed and sectioned in a chilled solution containing (in mM): choline chloride 110, KCl 2.5, NaH_2_PO_4_ 1.3, NaHCO_3_ 25.0, CaCl_2_ 0.5, MgCl_2_ 7, glucose 20, Na‐ascorbate 1.3, Na‐pyruvate 0.6. The solution was pre‐saturated with 95% O_2_ and 5% CO_2_. Horizontal slices (300 μm thickness) were prepared with a Leica VT1000S vibratome (Leica, Germany). Subsequently, slices were recovered at 34°C for 30 min in oxygenated artificial cerebrospinal fluid (ACSF) composed of (in mM): NaCl 125, KCl 2.5, NaH_2_PO_4_ 1.3, NaHCO_3_ 25, CaCl_2_ 2, MgCl_2_ 1.3, Na‐ascorbate 1.3, Na‐pyruvate 0.6, glucose 10, and then transferred to a recording chamber constantly perfused with the same ACSF (2 mL/min) at room temperature. Extracellular field potential recordings were made using microelectrodes (~1 MΩ) filled with ACSF. Evoked synaptic responses were triggered by perforant path stimulation with a concentric bipolar tungsten electrode (World Precision Instruments). LTP was induced with the HFS paradigm consisting of 100 pulses at 100 Hz, repeated with 20 s inter‐burst interval for four times. ACSF with additional 10 μm SCH23390 was used to trigger SCH23390‐mediated fEPSPs. All data were obtained using MultiClamp 700B patch‐clamp amplifiers (Molecular Devices), sampled at 5 kHz and low‐pass filtered at 2 kHz using a Digidata 1550B analog–digital interface (Molecular Devices, USA). All pharmacological agents were purchased from Sigma.

### 2.3 Stereotaxic viral injection

Stereotaxic viral injection was performed similarly as previously described.^25^ Adult mice were anesthetized with 1% pentobarbital sodium (35 mg/kg, i.p.) and head‐fixed in a stereotaxic device (68,025, RWD life science, China). Viruses were bilaterally injected (300 nL per side, 40 nL/min) with a glass pipette^26^ (tip size, ~20 μm) at the following coordinates relative to bregma: anteroposterior, −2.06 mm; dorsoventral, −2.0 mm; and mediolateral, ±1.5 mm. After injection, the glass pipette was left in place for 10 min before slowly removing it. The animals were subjected to behavioral tests 3 weeks after virus injection. Brains were sectioned afterward to verify the infusion sites. Data were excluded if GFP expression was misdirected.

### 2.4 BrdU injection

5′‐bromo‐2′‐deoxyuridine (BrdU; B5002, Sigma) in saline was administered i.p. (100 mg/kg) twice a day for 3 days. Animals were sacrificed 24 h after the last BrdU injection.

### 2.5 Behavioral analysis

The analysis was carried out by investigators unaware of the animal genotype and grouping information. All tests were performed during the light period, and all mice were handled at least 5 min twice a day for 3 days before all tests. Only 10–12‐week‐old males were used. No bodyweight, whisker number, and motor coordination differences between groups were found during behavioral analysis.

#### 2.5.1 Open‐Field Test (OFT)

The mice were gently placed at the center of a rectangular chamber (45 × 45 × 45 cm), and movement was monitored for 5 min using an automated video tracking system (Supermaze 2.0, Softmaze, China). After each trial, the apparatus was swept with 75% alcohol to avoid the presence of olfactory cues. The distance traveled during a session was measured.

#### 2.5.2 Radial 8‐arm maze (RAM)

**FIGURE 2:**
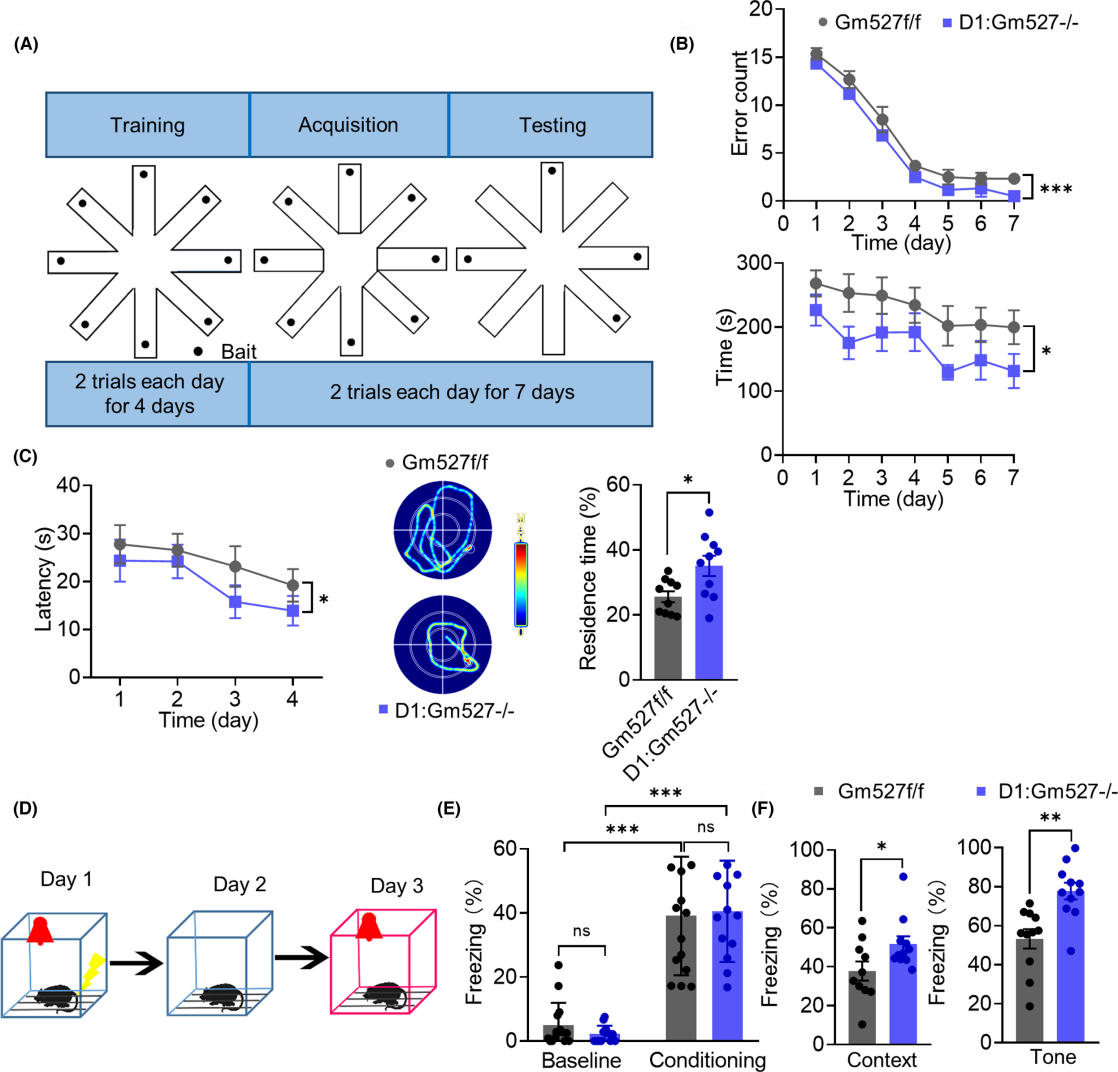
Working memory and long‐term memory are improved in D1:Gm527−/− mice. (A) Schematic diagram of the RAM experiment design. All the arms were opened and baited during the 4‐day training period and only four arms were constantly opened and baited during the following 7‐day testing period. Two trials were performed each day for both periods. (B) D1:Gm527−/− mice had fewer error arm entries and took less time to find all the baits (*n* = 13 and 15 for Gm527f/f and D1:Gm527−/− mice, respectively, two‐way ANOVA, *F* (1, 70) = 13.55, *p* < 0.0001 for error arm entries and *F* (1, 70) = 17.36, *p* = 0.016 for the total time). (C) Long‐term memory was improved in D1:Gm527−/− mice tested by MWM. The hidden platform was placed in a fixed position, and the mice were given four trials daily from four starting positions in the acquisition period to calculate the latency of reaching the platform. Then the platform was removed during the probe period, and the time spent in the target quadrant was measured. Representative images show the tracks to find the hidden platform (middle). Escape latency in the acquisition phase (left) was decreased, and the percentage of residence time in the probe phase (right) was increased in D1:Gm527−/− mice (*n* = 13 and 15 for Gm527f/f and D1:Gm527−/− mice, respectively; for escape latency, two‐way ANOVA, *F* (3, 217) = 2.711, *p* = 0.0460; for residence time percentage, unpaired *T*‐test, *p* = 0.015). (D) Schematic diagram of the fear conditioning experiment design. On day 1, mice were placed in context A with foot shock and tone. On day 2, mice were placed in context A without foot shock and tone to test contextual fear conditioning. On day 3, mice were placed in similar context B with tone but no foot shock to test tone‐cued fear conditioning. (E) Throughout the training, Gm527f/f and D1:Gm527−/− mice froze at comparable level during the trace interval (*n* = 13 and 15 for Gm527f/f and D1:Gm527−/− mice, respectively, two‐way ANOVA, *F* (1, 52) = 110.9, *p* < 0.0001). (F) Contextual and tone‐cued fear conditioning were improved in D1:Gm527−/− mice (*n* = 13 and 15 for Gm527f/f and D1:Gm527−/− mice, respectively, unpaired *T*‐test, *p* = 0.0419 and 0.0013 for contextual and tone‐cued fear conditioning).

The RAM consisted of a transparent organic plastic maze with eight equally spaced arms radiating from an octagonal central platform (Figure 2A). Before the training, the animals were kept on a restricted diet, and body weight was maintained at 85% of their free‐feeding weight with water available ad libitum. To train the mice to acclimatize the RAM, all the arms were baited, and mice were allowed to explore the maze where mice learned that only a single bait existed at the terminal end of the arms for 10 min twice a day for four consecutive days.

Then mice were tested two trials a day for 7 days, and each trial consisted of two parts: acquisition and testing. During acquisition, all the arms were baited with four constant opened arms and four constant closed arms. Mice were placed in the central area and allowed to explore the maze freely for 5 min. Then they were taken out and put back into the home cage for 3 min. During this period, the maze was thoroughly cleaned with 75% alcohol to eliminate the olfactory signal, and the four previously closed arms were opened. During testing, the mice were returned to the central area with all eight arms open. The number of errors (repeated entries into a previously visited arm or omission of an arm) and the total time to retrieve all pellets were scored. Trials were terminated after all pellets were retrieved or 10 min had elapsed.

#### 2.5.3 Morris Water Maze Test

Spatial learning and memory were evaluated in the Morris water maze (MWM) task as described.^27^ The MWM experimental setup consisted of a large circular pool (diameter: 180 cm) filled with water, maintained at 22–25°C and a depth of 60 cm, in a room with visual cues (triangle, square, cross, and circle) under soft and diffuse light conditions. The water was made opaque by adding nontoxic white paint (titanium dioxide). The pool was then virtually divided into four equal quadrants (NE, NW, SE, and SW) with four starting positions (N, S, E, and W). The platform was placed in the middle of one virtual quadrants throughout the training. In the acquisition phase, the animals were given four trials daily from four starting positions (1 min per mouse, with an interval of 1 h) for four consecutive days. For each trial, the latency to reach the platform was calculated. A probe trial was given on day 5 when the platform was removed. The time spent in the target quadrant was measured. Animals that did not swim or search for the goal were excluded from the analysis and further tests. Recording and analyzing behavior were carried out with a video tracking system (TMV‐100S, TaiMeng, China).

#### 2.5.4 Contextual and tone‐cued fear conditioning

The shock‐associated training context A and the similar context B without shock shared many features, including an exposed stainless‐steel grid floor and roof. The similar context B differed from training context A in that four plastic inserts were used to cover the walls. A nonalcoholic antiseptic solution was used to clean the grids between trials. On the first day, mice were allowed to explore the conditioning chamber (context A) (29 × 29 × 24 cm; H10‐11M‐TC, Coulbourn Instruments) for 180 s before the onset of a tone that lasted 30 s at 80 dB and 5 kHz. A foot shock (0.75 mA, 2 s) was administered during the last 2 s of the tone presentation and co‐terminated with the tone. The tone‐shock pairing was delivered three times, with an intertrial interval of 30 s. Mice were returned to the home cage after the conditioning session was completed. The freezing level of the last tone–shock pairing was measured to evaluate their learning. One day later, mice were placed into context A without shock and tone for 5 min to evaluate the contextual fear memory. On the third day, the tone‐cued fear memory was assessed by placing them into context B with a tone that lasted 30 s at 80 dB and 5 kHz but without shock. The freezing levels were measured by a video camera (Freezeframe, Coulbourn Instruments).

### 2.6 Immunofluorescence staining

Mice were anesthetized with 1% pentobarbital sodium (35 mg/kg, i.p.) and perfused transcardially with 0.1 M PBS followed by 4% paraformaldehyde in 0.1 M PBS. Brains were then removed and fixed overnight in 4% paraformaldehyde at 4°C. After dehydrating in 30% sucrose, brains were frozen in OCT medium (Tissue‐Tek, Sakura, Japan) and sliced into 30‐μm coronal sections using a cryostat (HM550, Thermo Scientific). After rinsing three times in PBS, brain sections were blocked in blocking buffer (PBS with 0.3% Triton X‐100 containing 10% goat serum and 3% BSA) for 60 min at room temperature. Sections were then incubated at 4°C overnight in blocking buffer containing the following primary antibodies: rabbit anti‐Ki67 (ab15580, Abcam; 1:1000), rat anti‐BrdU (ab74545, Abcam; 1:300). After washing with PBST (PBS with 0.05% Tween‐20) three times, sections were incubated with corresponding fluorophore‐conjugated secondary antibodies (goat anti‐rabbit IgG conjugated with Alexa Fluor 594, AS039, ABclonal, 1:500; goat anti‐rat IgG conjugated with Rhadomine, AS022, ABclonal, 1:500) in blocking buffer for 1 h at room temperature. After washing with PBST three times, sections were mounted on glass slides with mounting medium (containing DAPI, BL739A, Biosharp, China), and images were taken using Olympus Fluoview FV1000 (Olympus, Japan). Quantification of labeling was determined by counting all fluorescent cells in every sixth section. A total of five images were analyzed per mouse, and each group contained five mice.

For BrdU staining, sections were incubated with 2 N HCl for 30 min at 37°C to denature the DNA, followed by neutralization with 0.1 M borate buffer (pH 8.5) for 10 min at room temperature. After neutralization, sections were rinsed with PBST several times and incubated in blocking buffer for 60 min at room temperature before incubation with primary antibodies.

### 2.7 Sholl analysis

Sholl analysis was performed as previously described.^25^ The total dendritic branches in one GFP+ cell in the DG were scanned by an Olympus Fluoview FV1000 (Olympus, Japan). Dendritic branches were analyzed by the Image J Sholl Analysis Plugin (Figure S2). The center of all concentric circles was defined as the center of cell soma. Five mice in each group and five sections in each mouse were picked, 8–10 cells in the DG from each section were analyzed and counted.

### 2.8 Dissection of hippocampal dentate gyrus

The dentate gyrus was isolated as described previously.^28^ Mice were anesthetized with 1% pentobarbital sodium (35 mg/kg, i.p.), and the brain was dissected out from the skull and placed into ice‐cold PBS. In a Petri dish containing ice‐cold PBS, the brain was cut along the longitudinal fissure, and the regions posterior to lambda (midbrain, hindbrain, and cerebellum) were cut off. Then the cerebral hemisphere medial side was placed up, and the diencephalon was carefully removed under a dissection microscope to expose the medial side of the hippocampus. A sharp needle tip was inserted into each side of the DG and slid along the septo‐temporal axis of the hippocampus to isolate the DG. The DG from the other cerebral hemisphere was isolated using the same method.

### 2.9 Western blot

Tissue homogenates were prepared on ice in RIPA buffer (50 mM Tris–HCl, pH 7.4, 150 mM NaCl, 5% sodium deoxycholate, 1% NP40), 1 mM phenylmethylsulfonyl fluoride (PMSF), and 1 μg/mL protease inhibitor. The plasma membrane proteins (containing D1R) were obtained using a membrane protein extraction kit (BB31161, BestBio Biotechnology, China). Lysates were cleared by centrifugation at 13,000 *g* for 15 min. Protein concentration of supernatants was determined using BCA Protein Assay Kit (P0012, Beyotime Biotechnology, China). Homogenates and the plasma membrane proteins were resolved on SDS/PAGE and transferred to PVDF membranes, which were then incubated in blocking buffer (TBS containing 0.1% Tween‐20 and 5% BSA) for 1 h at room temperature before adding the primary antibodies for incubation overnight at 4°C. After washing with TBST (TBS with 0.1% Tween‐20), the membranes were incubated with horseradish peroxidase‐conjugated secondary antibodies (BL003A, Biosharp, China; 1: 10,000) in TBST for 1 h at room temperature. Immunoreactive bands were visualized using enhanced chemiluminescence (RM00020, ABclonal, China). Films were scanned using MicroChemi 4.2 (DNR Bio‐imaging Systems, Israel). Primary antibodies used were: rabbit anti‐CREB (AF6188, Affinity; 1: 1000), rabbit anti‐phosphate CREB (pCREB) (AF6189, Affinity; 1: 1000), rabbit anti‐GluN2A (A0924, Abclonal; 1: 1000), rabbit anti‐GluN2B (ab65783, Abcam; 1:1000), rabbit anti‐Na+/K+‐ATPase (A11683, Abclonal; 1:1000), rat anti‐D1R (D2944, Sigma; 1:1000), rabbit anti‐Gm527 (WG03611, Abclonal; 1: 500), and rabbit anti‐α tubulin (AC003, Abclonal; 1: 4000). The band density was measured by Image J software, and data were analyzed by GraphPad Prism 8.0 (GraphPad Software, USA). The original western blot images were shown in Appendix S1.

### 2.10 Immunoprecipitation

Tissue homogenates were prepared on ice in RIPA buffer (50 mM Tris–HCl, pH 7.4, 150 mM NaCl, 5% sodium deoxycholate, 1% NP40), 1 mM phenylmethylsulfonyl fluoride (PMSF), and 1 μg/mL protease inhibitor. Lysates were cleared by centrifugation at 13,000 *g* for 15 min. Protein concentration of supernatants was determined using BCA Protein Assay Kit (P0012, Beyotime Biotechnology, China), which was diluted to 1 μg/μL. D1R antibody (D2944, Sigma) was added to the diluted sample solution and incubated overnight at 4°C. Protein A/G agarose beads (16‐266, Millipore) were mixed into the sample solution that was incubated with the antibody overnight and then incubated for 2–4 h at 4°C to pair the antibody with the beads. After the immunoprecipitation reaction, beads were centrifuged to the bottom of the tube at 3300 *g* for 2 min at 4°C. Then beads were rinsed with PBS at 4°C for three times and incubated with PBS and 2 × SDS buffer at 95°C for 5 min. Samples were loaded to 10% SDS‐PAGE gel, and western blot was performed.

### 2.11 Quantitative real‐time PCR (qRT‐PCR)

Total RNAs were extracted from tissues using Universal Total RNA Isolation Reagent (BS259A, Biosharp, China). Reverse transcription reaction was performed with HiScript II Q RT SuperMix for qPCR (+gDNA wiper) (R223, Vazyme, China). The primers of Gm527 gene (NM_217648) used for qRT‐PCR were forward squences 5′‐CTGGAAGGCGCCACTTTATT‐3′ and reverse squences 5′‐GCAGGCGGGAGAACAATTTA‐3′. Taq Pro Universal SYBR qPCR Master Mix (Q712, Vazyme, China) was used, and qRT‐PCR program was carried out in Quantagene q225 real‐time PCR system (Kubo Technology, China). GAPDH (NM_017008) was used as the control. All reactions were run in triplicate. The transcript level of Gm527 was analyzed according to the relative quantification method.^29^

### 2.12 Fluorescence in situ hybridization

To assess the expression of Gm527 in D1:Gm527−/− mice, we performed hybridization chain reaction (HCR)^30^, ^31^ of in situ hybridization for labeling Gm527 and D1R mRNA in tissue slices. First, we designed 28 probes for D1R and 12 probes for Gm527 using a custom‐written software (available at https://github.com/GradinaruLab/HCRprobe). Each probe consists of 20‐nt target sequence, 2‐nt spacer, and 18‐nt initiator (Table S1). Then we blasted full sequences (target sequences with spacer and initiator and calculate Smith–Waterman alignment scores between all possible pairs to exclude probes forming cross‐dimers). The designed probes were synthesized by AuGCT Biotechnology. HCR was performed on 30 μm‐thick slices and kept in RNase‐free solution (BL510B, Biosharp, China). Slices were permeabilized in 70% ethanol at 4°C for 12–16 h and further permeabilized in PBST (1 × PBS with 0.1% Triton X‐100) for 1 h at 37°C. Pre‐hybridized in 30% hybridization buffer (5 × saline‐sodium citrate (SSC), 30% formamide, 9 mM citric acid, PH 6.0, 0.1% Tween 20, 50 μg/mL heparin, 1 × Denhardt's dolution, 10% dextran sulfate) for 10 min at 37°C. Then the samples were incubated in pre‐warmed hybridization buffer including probes (10 pmol for each) at 37°C for 3 h. After hybridization, we performed stringent washes with 30% probe wash buffer (5 × SSC, 30% formamide, 9 mM citric acid, PH 6.0, 0.1% Tween 20, 50 μg/mL heparin) for 30 min at 37°C and washes with 5 × SSCT (5 × SSC with 0.1% Tween 20) for 10 min at room temperature. The amplification step was performed as described^32^ overnight. Briefly, pre‐amplification in amplification buffer (5 × SSC, 0.1% Tween 20, 10% dextran sulfate) in a humidified chamber for 30 min at room temperature. Then the samples were incubated in pre‐warmed amplification buffer with 6 pmol of each fluorescently labeled hairpin (heat at 95°C for 90 s and cool‐to‐room temperature in a dark drawer for 30 min) for 12–16 h at room temperature. After amplification, hairpins were removed by incubating slide in 5 × SSCT at room temperature. After washing with 5× SSCT three times, samples were mounted on glass slides with mounting medium (containing DAPI, BL739A, Biosharp, China), and images were taken using Olympus Fluoview FV1000 (Olympus, Japan). The number of Gm527 dots in the areas of co‐localization of DAPI with D1R was calculated in 6–8 images in two sections for each mouse, and the result was presented as the number of Gm527 dots/the total area of co‐localization with DAPI and D1R.

### 2.13 Statistical analysis

All statistical analysis was performed using GraphPad Prism 8.0 (GraphPad Software, USA). The normal distribution test was performed using the Shapiro–Wilk test. Data followed a normal distribution and were analyzed using two‐tailed Student's *T*‐test, one‐way ANOVA followed by Tukey's post hoc test, or two‐way ANOVA with Bonferroni post hoc test. Otherwise, data were analyzed using the Mann–Whitney test. Statistical differences were considered to be significant when *p <* 0.05.

## 3 RESULTS

### 3.1 Improvement of schizophrenia‐related cognitive behaviors in D1:Gm527−/− mice

It has been reported that C14orf28 might be a schizophrenia‐related gene,^23^ and its rodent homologous Gm527 binds to D1R directly, tested by yeast two‐hybrid experiments.^24^ To investigate the expression of Gm527 on the MK801‐induced schizophrenia model, MK801 (HY‐15084, MCE, 0.5 mg/kg) or saline (control) was administered intraperitoneally (i.p.) twice a day for 5 days. We confirmed that Gm527 was a DRIP of D1R with immunoprecipitation (Figure 1A), and its expression was dramatically increased in MK801‐injected mice hippocampus and PFC (Figure 1B), consistent with the observation of C14orf28 overexpression in schizophrenia patients.

The murine Gm527 gene is located on chromosome 12, spans about 6 kb, and is divided into 5 exons (Figure 1C). To gain insight into the function of Gm527 in dopamine D1R‐positive neurons, a conditional knockout line of Gm527 (Gm527‐floxed) was generated by insertion of two loxP sites flanking exon 2 that contained start codon and crossed with D1‐Cre mice expressing Cre under the control of D1 gene (Figure 1C). Knockout Gm527 in D1‐Cre:Gm527‐floxed f/f (D1:Gm527−/−) mice was confirmed by the decreased Gm527 transcript and protein levels in the DG, but not in the PFC (Figure 1D,E). In addition, fluorescence in situ hybridization (FISH) demonstrated the D1R‐positive cells were Gm527‐positive as well in the DG of Gm527 f/f mice; however, the D1R cells were still positive in the DG of D1:Gm527−/− mice while Gm527 were not (Figure 1F). And the expression of Gm527 mRNA was ablated in D1R‐positive cells in the DG of D1:Gm527−/− mice (Figure 1G).

To investigate whether knockout Gm527 could induce schizophrenia‐related behaviors, we first evaluated locomotion hyperactivity with open field test (OFT), a characteristic rodent phenotype that related to the psychomotor agitation of schizophrenic patients,^33^, ^34^, ^35^ and hyperactivity has been reported in mice lacking the D1R.^36^, ^37^ However, the total distance traveled was not changed in D1:Gm527−/− mice compared with the controls (Figure S1a,b).

Second, we evaluated the effect of Gm527 knockout in D1R‐positive neurons on working memory using the radial eight‐arm maze test (RAM) (Figure 2A). Working memory deficit is the central phenotype in schizophrenia, resulting from dopaminergic dysfunction.^38^, ^39^ A significant trial effect was observed in the total number of wrong entries (repeated entries into a previously visited arm or omission of an arm) and the total time to retrieve all pellets, indicating that both groups were able to improve their performance to retrieve baits. Surprisingly, compared with the control, D1:Gm527−/− mice significantly decreased the wrong entries and the total time to retrieve all the baits (Figure 2B), suggesting improvements in working memory. It has been well established that working memory can be enhanced by D1R stimulation^40^ and impaired by D1R antagonist,^41^ implying that the D1R signaling pathway might be promoted by Gm527 knockout.

Third, we examined the long‐term memory by Morris water maze (MWM) and fear conditioning. Long‐term memory was also affected in schizophrenia patients, especially in patients with hippocampal dysfunction.^42^ In the acquisition phase of MWM, the animals were given four trials daily for four consecutive days. The significant trial effect was also observed in the escape latency of both groups, however, the D1:Gm527−/− mice learned the task faster in the acquisition phase (Figure 2C). In the probe trial phase (day 5), the D1:Gm527−/− mice spent more time in the target quadrant than the Gm527f/f ones (Figure 2C). For the fear conditioning test, mice were allowed to explore the conditioning chamber for 180 s before the onset of a tone paired with foot shock three times in context A for the first day. The freezing levels in the third tone‐shock pairing conditioning phase were comparable between Gm527f/f and D1:Gm527−/− mice (Figure 2D,E). The next day, they were exposed to context A without tone and foot shock to observe contextual fear conditioning. On the third day, the freezing level was observed in a similar context B with tone cue but no foot shock to observe tone‐cued fear conditioning. As in MWM, both contextual and tone‐cued fear conditioning were increased in D1:Gm527−/− mice (Figure 2F), suggesting that the D1:Gm527−/− mice enhanced long‐term memory, possibly due to the changes in synaptic plasticity.

### 3.2 Newborn cells increase in the DG of D1:Gm527−/− mice

**FIGURE 3:**
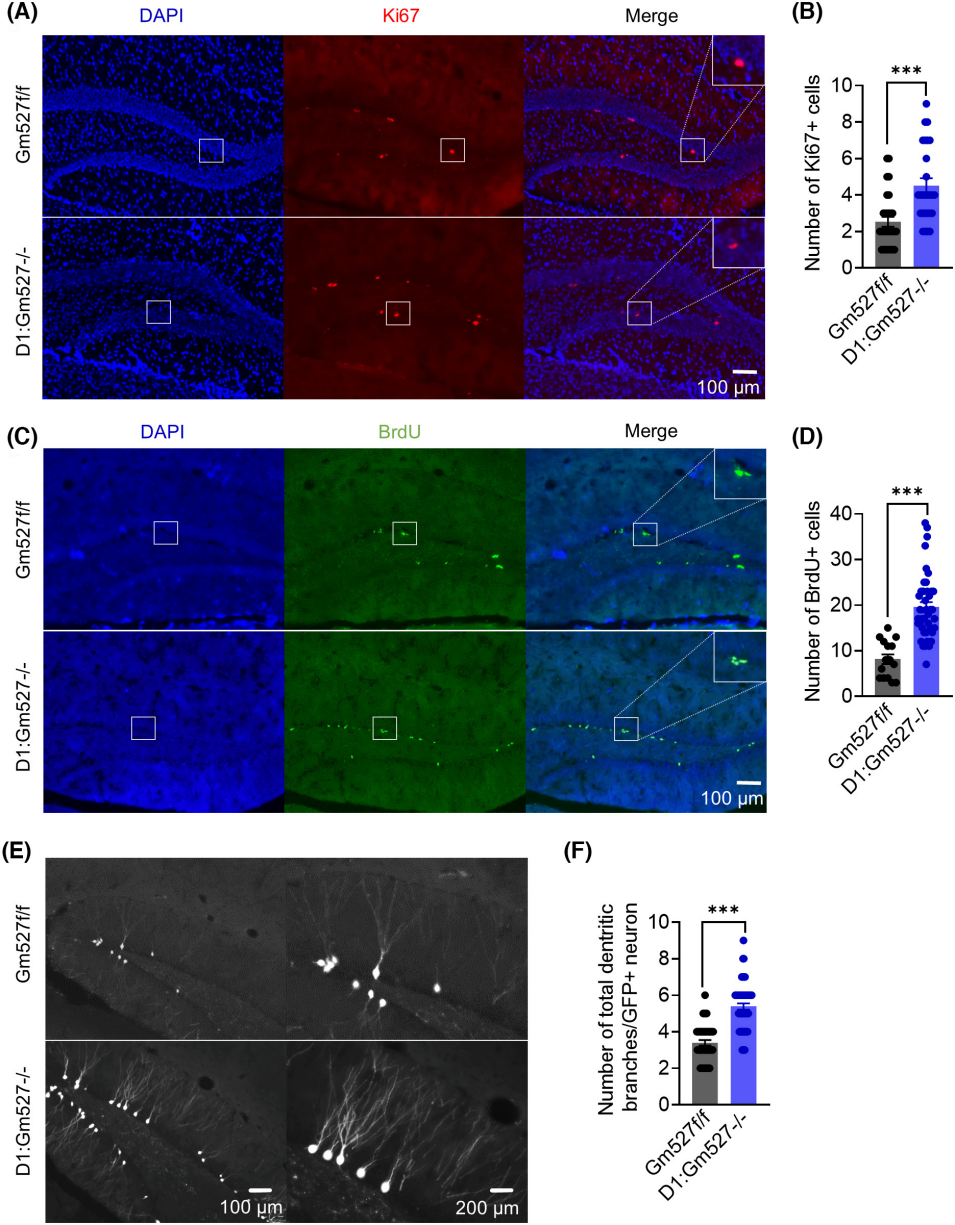
Increased adult neurogenesis in the DG of D1:Gm527−/− mice. (A) Representative photomicrographs showing Ki67+ and DAPI+ cells in the DG. (B) The number of Ki67+ cells in the DG increased in D1:Gm527−/− mice (five mice per group and five sections per mouse, Mann–Whitney test, *p* < 0.0001). (C) Representative photomicrographs showing BrdU+ and DAPI+ cells in the DG. (D) The number of BrdU+ cells in the DG increased in D1:Gm527−/− mice (five mice per group and five sections per mouse, Mann–Whitney test, *p* < 0.0001). (E) Representative images of the dendritic branches of GFP+ cells in the DG. GFP+ cells were labeled with retrovirus pROV‐EF1a‐EGFP, which was injected in the DG. (F) The total number of dendritic branches of one GFP+ neurons increased in D1:Gm527−/− mice (three mice per group, five sections per mouse, and 8–10 cells per section, Mann–Whitney test, *p* < 0.0001).

Adult hippocampal neurogenesis is crucial in context discrimination in spatial memory, and its dysregulation is related to schizophrenia.^43^ Consistent with the improved cognitive behaviors, D1:Gm527−/− mice showed increased neural stem cell proliferation by Ki67 (Figure 3A,B) and BrdU immunostaining (Figure 3C,D) in the DG of D1:Gm527−/− mice.

To further elucidate the maturation of NSCs in the DG, newborn neurons were infected by retrovirus pROV‐EF1a‐EGFP, and the total dendritic branches of GFP‐positive neurons were analyzed by Sholl analysis (Figure S2). A significant increase of total dendritic branches in D1:Gm527−/− mice also suggested that knockout Gm527 in D1R‐positive neurons promoted NSCs maturation (Figure 3E,F).

### 3.3 D1R expression on the plasma membrane is increased in the DG of the D1:Gm527−/− mice, enhancing LTP through cAMP pathway and NMDA receptor

**FIGURE 4:**
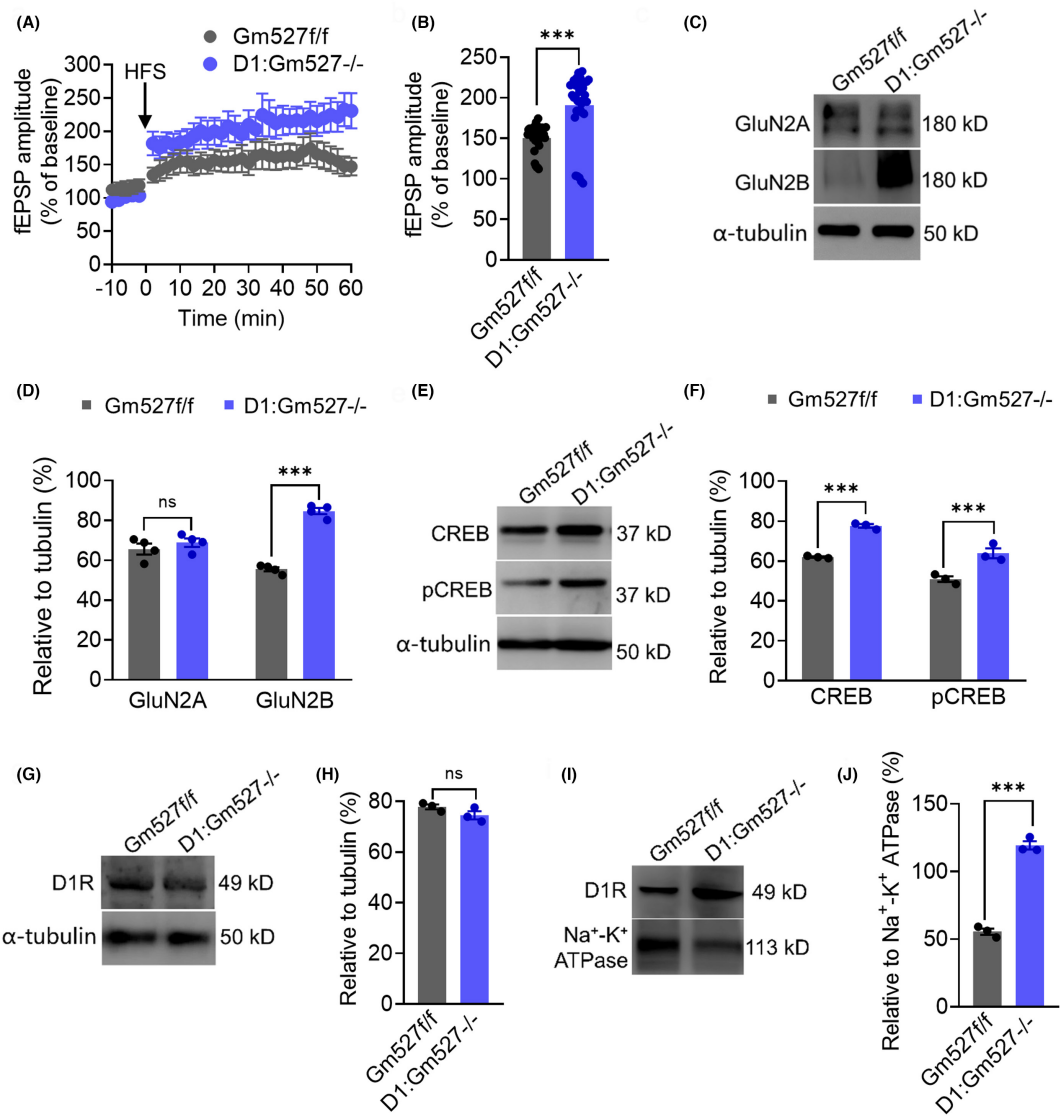
Synaptic plasticity is enhanced in the DG of D1:Gm527−/− mice. (A) HFS induced LTP was enhanced in the DG of D1:Gm527−/− mice. (B) Quantitative analysis of data in (A) (*n* = 4–5 mice/group, Mann–Whitney test, *p* < 0.0001). (C) The expression of GluN2B was increased in the DG of D1:Gm527−/− mice, whereas the expression of GluN2A was unchanged. (D) Quantification of GluN2A and GluN2B expressions in (C) (*n* = 3–4 mice/group, two‐way ANOVA, *F* (1, 12) = 66.66, *p <* 0.0001). (E) The expressions of CREB and pCREB were increased in the DG of D1:Gm527−/− mice. (F) Quantification of CREB and pCREB expressions in (E) (n = 3–4 mice/group, two‐way ANOVA, *F* (1, 8) = 90.61, *p* < 0.0001). (G) The expression of D1R was unchanged in the DG of D1:Gm527−/− mice. (H) Quantification of D1R expression in (G) (*n* = 3–4 mice/group, unpaired *T*‐test, *p* = 0.1564). (I) The expression of D1R was increased on the plasma membrane in the DG of D1:Gm527−/− mice. (J) Quantification of D1R expression in (I) (*n* = 3–4 mice/group, unpaired *T*‐test, *p* < 0.001).

Previous results revealed that spatial long‐term memory performances were enhanced in D1:Gm527−/− mice, we next investigated whether long‐term synaptic plasticity was affected in the DG. As shown in Figure 4A,B, high‐frequency stimulation (HFS) delivered to the perforant pathway (PP) consistently induced long‐term enhancement of synaptic transmission to dentate granule cells (DGCs). Evoked field excitatory postsynaptic potentials (fEPSPs) persisted a ~30% LTP enhancement in D1:Gm527−/− mice compared with the controls. Glutamatergic and dopaminergic afferents activation is required to induce LTP in the hippocampus,^44^ through reciprocal activation of NMDA receptor and D1R activation.^45^ To further investigate whether NMDA receptors were affected, we observed the expression of GluN2A and GluN2B, two subunits of the NMDA receptor. GluN2B interacts with calcium/calmodulin‐dependent protein kinase II (CaMKII), which is more important for plasticity at the postsynaptic density.^46^ Consistent with the literature, GluN2B but not GluN2A in the DG was significantly increased in D1:Gm527−/− mice (Figure 4C,D), suggesting that Gm527 knockout in D1R‐positive neurons might increase long‐term plasticity through NMDA receptor.

cAMP response element‐binding protein (CREB) activation is required for long‐term synaptic plasticity and memory consolidation,^47^ and we found a significant increase of CREB and pCREB protein in the DG of D1:Gm527−/− mice (Figure 4E,F).

To elucidate the mechanism of cAMP pathway activation, we investigated whether knockout Gm527 in the D1R‐positive neuron could affect D1R cell surface expression. The analysis revealed the D1R was unchanged in the DG of D1:Gm527−/− mice, whereas the cell surface D1R was increased, indicating that more D1R was transformed from the cytosol to the cell surface in the D1:Gm527−/− mice (Figure 4G–J).

Furthermore, the enhancement of LTP due to the D1R activation on the cell surface caused by knocking out Gm527 in D1R‐positive neurons was blocked by using SCH23390, a D1R antagonist (Figure S3).

### 3.4 Reversing Gm527 expression in the DG of D1:Gm527−/− mice causes memory deficits and reduced neurogenesis

**FIGURE 5:**
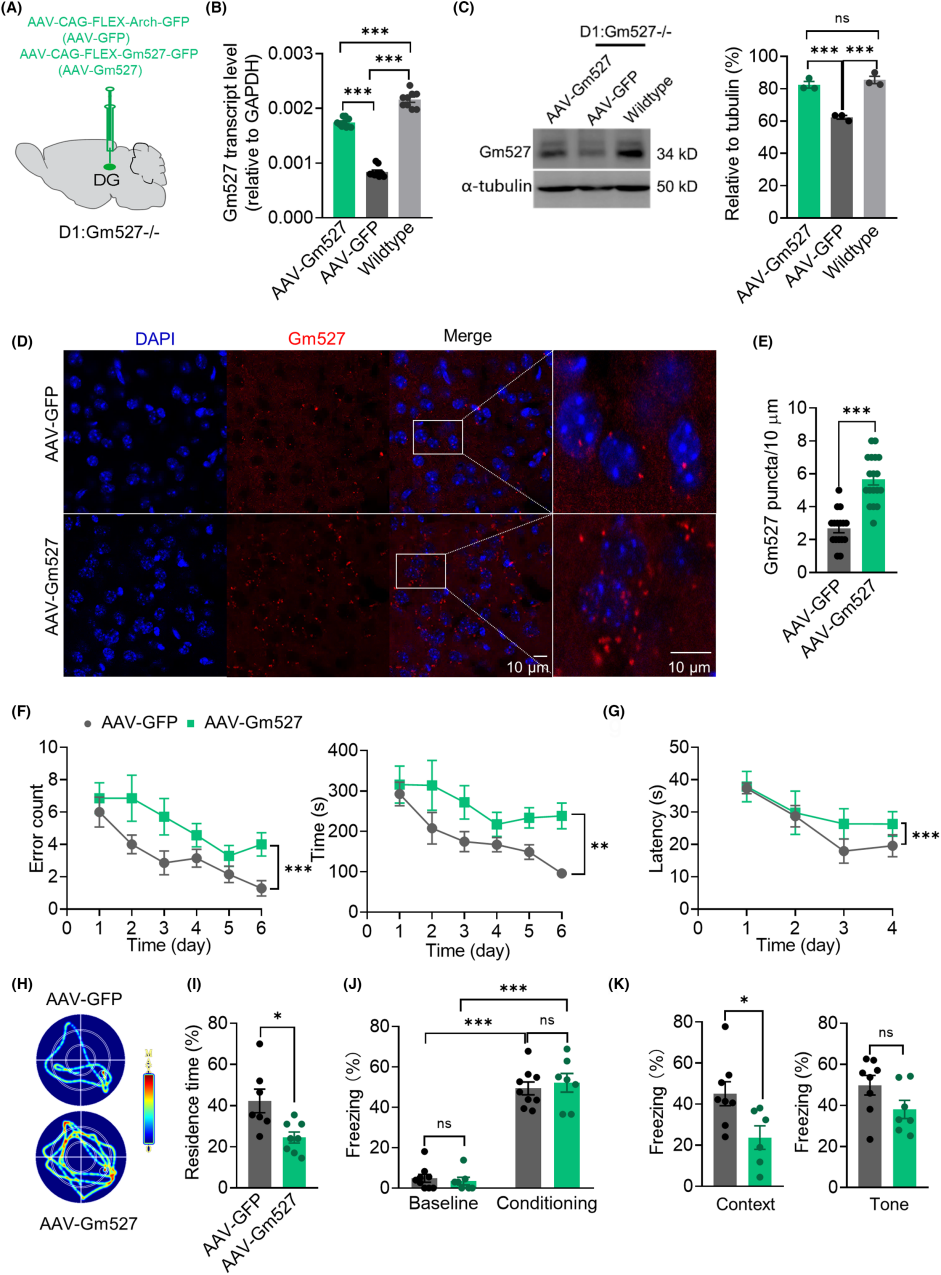
Reversing Gm527 expression in the DG of D1:Gm527−/− mice causes memory deficits. (A) Schematic of bilateral stereotaxic injection of AAV‐CAG‐FLEX‐Arch‐GFP (AAV‐GFP) or AAV‐CAG‐FLEX‐Gm527‐GFP (AAV‐Gm527) into the DG of D1:Gm527−/− mice. (B) Gm527 transcript level was increased after AAV‐Gm527 injection into the DG of D1:Gm527−/− mice (*n* = 3 mice/group in triplicate, one‐way ANOVA, *F* = 311.3, *p* < 0.0001). (C) Left, the expression of Gm527 was reversed to the level of wild‐type mice after AAV‐Gm527 injection into the DG of D1:Gm527−/− mice. Right, quantification of Gm527 expression in (C) (*n* = 3–4 mice/group, one‐way ANOVA, *F* = 44.56, *p* = 0.0003). (D) Representative images showing that the Gm527 mRNA in the DG of the AAV‐Gm527‐injected mice, tested by FISH. Gm527 mRNA, was detected by hybridization chain reaction (HCR) that simultaneously used Alexa546‐conjugated hairpin DNA for probe‐hairpin pair. (E) The expression of Gm527 mRNA in the DG increased in AAV‐Gm527‐injected mice (five mice per group and five sections per mouse, unpaired *T*‐test, *p* < 0.0001). (F) Working memory was impaired in AAV‐Gm527‐injected mice. AAV‐Gm527‐injected mice had more error arm entries and took more time to find all the baits (*n* = 7 for two groups, respectively, two‐way ANOVA, *F* (5, 72) = 6.575, *p* < 0.0001 for error arm entries and *F* (5, 72) = 4.526, *p* = 0.0012 for the total time). (G–I) Long‐term memory was impaired in AAV‐Gm527‐injected mice tested by MWM. (G) Escape latency in the acquisition phase was increased (*n* = 8 and *n* = 7 for AAV‐GFP and AAV‐Gm527 mice, respectively, two‐way ANOVA, *F* (3, 52) = 6.484, *p* = 0.0008). (H) Representative images showing the tracks to find the hidden platform. (I) The percentage of residence time in the probe trial phase was decreased in AAV‐Gm527‐injected mice (*n* = 8 and *n* = 7 for AAV‐GFP and AAV‐Gm527 mice, respectively, unpaired *T*‐test, *p* = 0.0114). (J) Throughout the training, AAV‐GFP and AAV‐Gm527‐injected mice froze at comparable level during the trace interval (*n* = 8 and *n* = 7 for AAV‐GFP and AAV‐Gm527, respectively, two‐way ANOVA, *F* (1, 28) = 236.5, *p* < 0.0001). (K) Contextual fear conditioning was impaired, but tone‐cued fear conditioning was unchanged in AAV‐Gm527‐injected mice (*n* = 8 and *n* = 7 for AAV‐GFP and AAV‐Gm527 mice, respectively, unpaired *T*‐test, *p* = 0.0258 and *p* = 0.0945 for contextual and tone‐cued fear conditioning).

To investigate whether the DG in adult D1:Gm527−/− mice is critical for memory enhancement, mice were stereotaxically injected with adeno‐associated virus AAV‐FLEX‐Arch‐GFP (AAV‐GFP) or AAV‐CAG‐FLEX‐Gm527‐GFP (AAV‐Gm527) into the DG of D1:Gm527−/− mice (Figure 5A). The transcript and protein levels of Gm527 were reversed to the equivalent level of wildtype mice after AAV‐Gm527 injection into the DG of D1:Gm527−/− mice (Figure 5B,C). In addition, FISH demonstrated the expression of Gm527 mRNA increased in the DG of AAV‐Gm527‐injected mice as well (Figure 5D,E).

Working memory was decreased after restoring the expression of Gm527 in the DG, revealed by more times of wrong entries and lengthened time to find the baits in the RAM test (Figure 5F). Long‐term memory was also decreased, revealed by lengthened time to escape and less time in the target quadrant in the MWM test (Figure 5G–I), and less freezing time in the contextual fear conditioning, whereas the freezing time in the tone‐cued fear conditioning was unchanged (Figure 5J,K). Similarly, the exploratory behavior was not affected after restoring Gm527 expression in the DG (Figure S1c,d). These results suggest that the DG is crucial for the working memory and long‐term memory improvement in the D1:Gm527−/− mice after Gm527 knockout.

**FIGURE 6:**
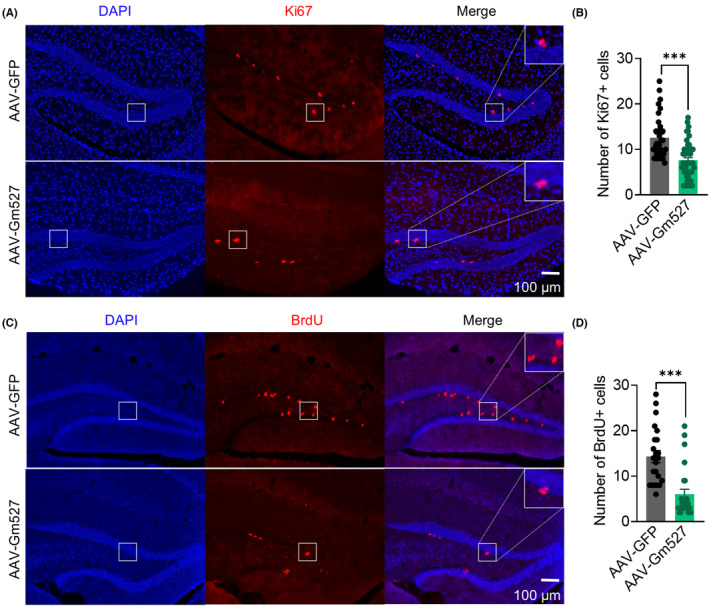
Decreased adult neurogenesis after reversing Gm527 in the DG. (A) Representative photomicrographs showing Ki67+ and DAPI+ cells in the DG. (B) The number of Ki67+ cells in the DG decreased in AAV‐Gm527‐injected mice (five mice per group and five sections per mouse, Mann–Whitney test, *p* < 0.0001). (C) Representative photomicrographs showing BrdU+ and DAPI+ cells in the DG. (D) The number of BrdU+ cells in the DG decreased in AAV‐Gm527‐injected mice (five mice per group and five sections per mouse, Mann–Whitney test, *p* < 0.0001).

Consistent with memory decrease after AAV‐Gm527 injection, NSCs proliferation in the DG was also decreased, revealed by Ki67 and BrdU staining (Figure 6A–D), suggesting that Gm527 re‐expression might inhibit adult neurogenesis in the DG, which might subsequently suppress context discrimination in spatial memory.

### 3.5 Reversing Gm527 expression in the DG of D1:Gm527−/− mice leads to synaptic plasticity deficit

**FIGURE 7:**
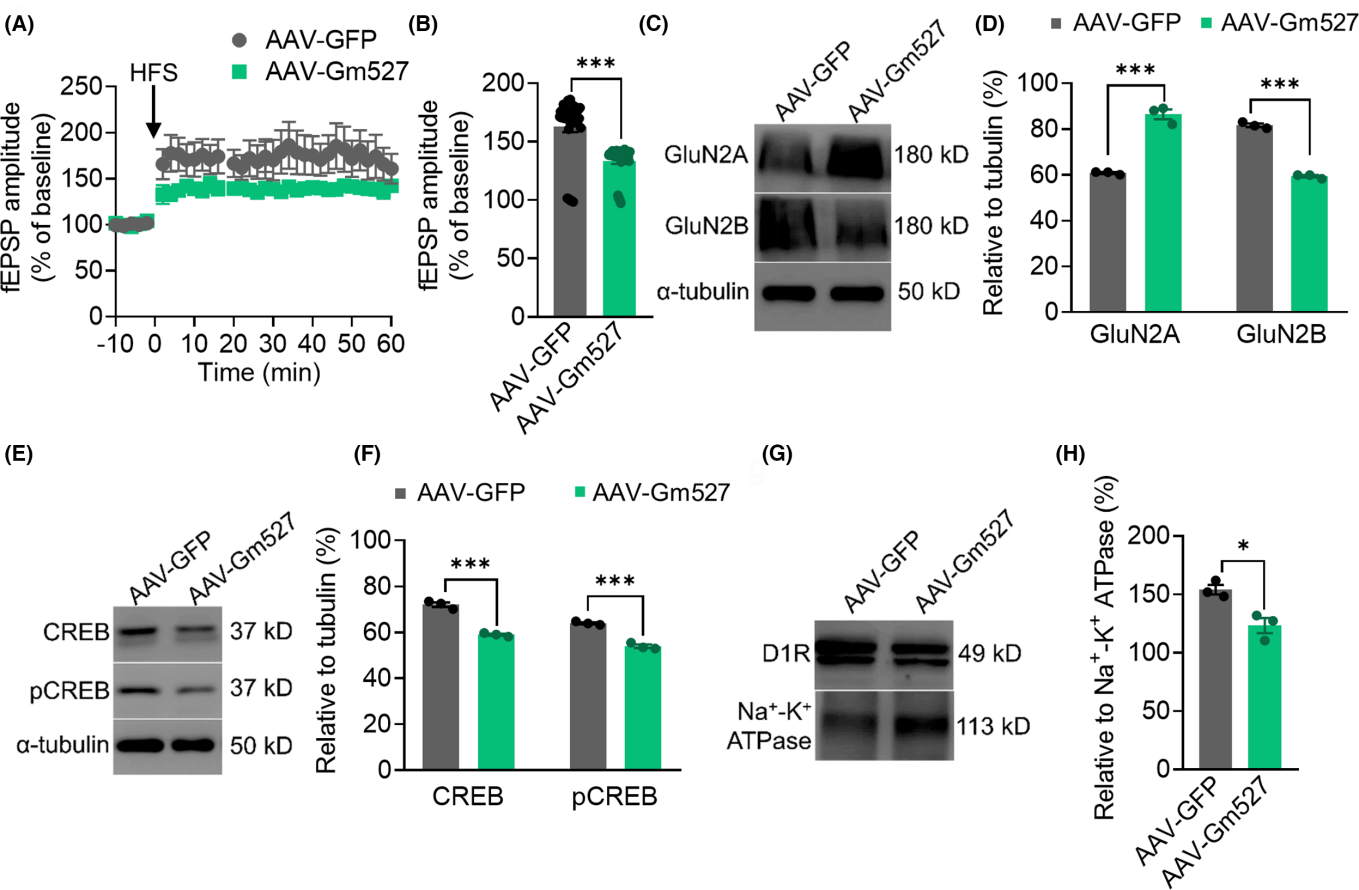
Synaptic plasticity is weakened in the DG of the AAV‐Gm527‐injected mice. (A) HFS induced LTP was weakened in the DG of AAV‐Gm527‐injected mice. (B) Quantitative analysis of data in (A) (*n* = 4–5 mice per group, Mann–Whitney test, *p* < 0.0001). (C) The expression of GluN2B was decreased in the DG of AAV‐Gm527‐injected mice, whereas the expression of GluN2A was increased. (D) Quantification of GluN2A and GluN2B expressions in (C) (*n* = 3–4 mice/group, two‐way ANOVA, *F* (1, 8) = 395.1, *p* < 0.0001). (E) CREB and pCREB expressions were decreased in the DG of AAV‐Gm527‐injected mice. (F) Quantification of CREB and pCREB in (E) (*n* = 3–4 mice/group, two‐way ANOVA, *F* (1, 8) = 267.9, *p* < 0.0001). (G) The expression of D1R was decreased on the plasma membrane in the DG of AAV‐Gm527‐injected mice. (H) Quantification of D1R expression in (G) (*n* = 3–4 mice/group, unpaired *T*‐test, *p* < 0.05).

Previous research revealed that spatial long‐term memory performances were weakened in AAV‐Gm527‐injected mice, we next investigated whether long‐term synaptic plasticity was affected in the DG. As shown in Figure 7A,B, fEPSPs persisted a ~20% LTP decline in AAV‐Gm527‐injected mice.

Consistent with LTP, the expression level of GluN2B was decreased in the AAV‐Gm527 injection group; however, GluN2A expression was increased (Figure 7C,D), suggesting that GluN2B might be more critical in synaptic plasticity in the DG. A significant decrease in CREB and pCREB was also observed after AAV‐Gm527 injection, which might underlie the LTP and long‐term memory decrease (Figure 7E,F). To investigate whether reverse of Gm527 could affect D1R cell surface expression, we tested the plasma membrane D1R protein expression by western blot assay. The analysis revealed the D1R was downregulated in AAV‐Gm527‐injected mice (Figure 7G,H).

### 3.6 Knockdown of Gm527 expression in the DG of D1‐Cre mice improves memory

**FIGURE 8:**
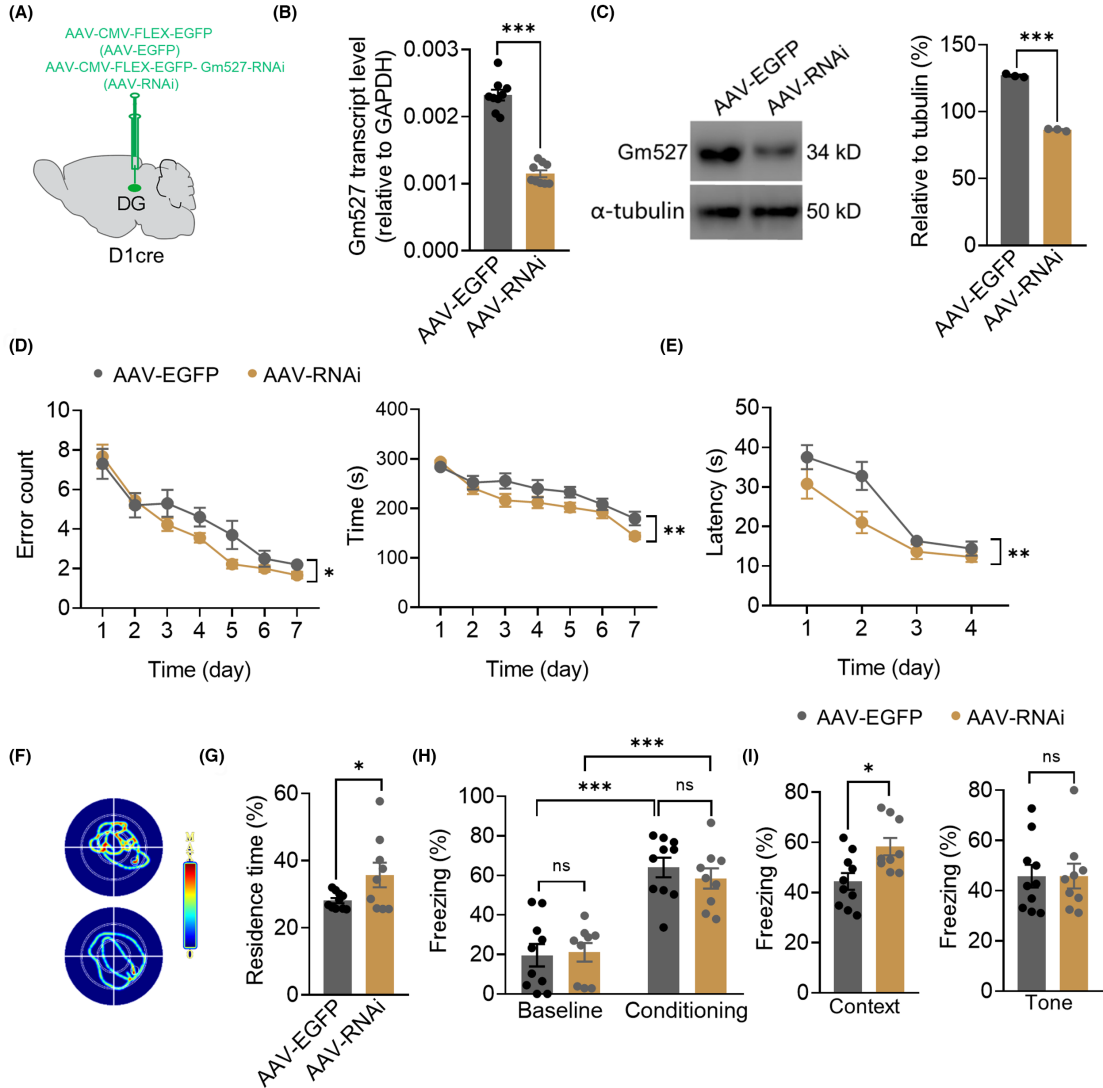
Knockdown Gm527 expression in the DG of D1‐Cre mice improves memory. (A) Schematic of bilateral stereotaxic injection of AAV‐CMV‐FLEX‐EGFP (AAV‐EGFP) or AAV‐CMV‐FLEX‐EGFP‐Gm527‐RNAi (AAV‐RNAi) into the DG of D1‐Cre mice. (B) Gm527 transcript level was decreased in the DG of AAV‐RNAi‐injected mice (*n* = 3 mice/group in triplicate, unpaired *T*‐test, *p* < 0.0001). (C) Left, Gm527 protein level was decreased in the DG of AAV‐RNAi‐injected mice. Right, quantification of Gm527 protein level (*n* = 3–4 mice/group, unpaired *T*‐test, *p* < 0.0001). (D) AAV‐RNAi‐injected mice had fewer error arm entries and took less time to find all the baits (*n* = 10 and 9 for AAV‐EGFP and AAV‐RNAi group, respectively, two‐way ANOVA, *F* (1, 119) = 4.790, *p* = 0.0306 for error arm entries and *F* (1, 119) = 10.84, *p* = 0.0013 for the total time). (E–G) Long‐term memory was impaired in AAV‐Gm527‐injected mice tested by MWM. (E) Escape latency in the acquisition phase was decreased in AAV‐RNAi‐injected mice (*n* = 10 and 9 for AAV‐EGFP and AAV‐RNAi group, respectively, two‐way ANOVA, *F* (1, 68) = 10.26, *p* = 0.0021). (F) Representative images show the tracks to find the hidden platform. (G) The percentage of residence time in the probe phase was increased in AAV‐RNAi‐injected mice (*n* = 10 and 9 for AAV‐EGFP and AAV‐RNAi group, respectively, unpaired *T*‐test, *p* = 0.0471). (H) Throughout the training, AAV‐EGFP and AAV‐RNAi‐injected mice froze at comparable levels during the trace interval (*n* = 10 and 9 for AAV‐EGFP and AAV‐RNAi group, respectively, two‐way ANOVA, *F* (1, 34) = 62.85, *p* < 0.0001). (I) Contextual and tone‐cued fear conditioning were improved in AAV‐RNAi‐injected mice (*n* = 10 and 9 for AAV‐EGFP and AAV‐RNAi group, respectively, unpaired *T*‐test, *p* = 0.0105 and 0.9915 for contextual and tone‐cued fear conditioning).

To further investigate the role of Gm527, mice were stereotaxically injected with AAV‐CMV‐FLEX‐EGFP (AAV‐EGFP) or AAV‐CMV‐FLEX‐EGFP‐Gm527‐RNAi (AAV‐RNAi) into the DG of D1‐Cre mice (Figure 8A). Knockdown Gm527 in D1‐Cre mice was confirmed by the decreased Gm527 transcript and protein levels in the DG (Figure 8B,C). Gm527 knockdown in the DG also did not affect the locomotion (Figure S1e,f); however, working memory was improved, revealed by less times of wrong entries and less time to find the baits in the RAM test (Figure 8D). Long‐term memory was also improved, revealed by shortened time to escape and lengthened time in the target quadrant in the MWM test (Figure 8E–G), and more freezing time in the contextual fear conditioning, whereas the freezing time in the tone‐cued fear conditioning was unchanged (Figure 8H,I). This suggested that Gm527 is crucial for the working memory and long‐term memory improvement in the D1:Gm527−/− mice.

## 4 DISCUSSION

Our study confirms that Gm527 downregulates D1R expression on the plasma membrane and its knockout in D1R‐positive neurons promotes memory and adult neurogenesis through cAMP pathway activation in the DG. First, Gm527 was overexpressed in MK801‐injected mice (Figure 1B), and knockout Gm527 in D1R positive neurons improved working memory, long‐term memory, and adult neurogenesis in the DG (Figures 2, 3). To further investigate the mechanism, we found that the synaptic plasticity of glutamate neurons (Figure 4A,B) in the DG was increased, which might be due to activation of the cAMP pathway by upregulating D1R plasma membrane expression in the DG after knockout Gm527 (Figure 4C–J). The function of Gm527 in the DG was further confirmed by AAV‐Gm527 local injection to the DG of D1:Gm527−/− mice to reverse Gm527 expression or AAV‐RNAi local injection to the DG of D1‐Cre mice to knockdown Gm527 expression in D1R‐positive neurons (Figures 5, 6, 7, 8).

Dopamine receptor‐interacting proteins (DRIPs) constitute a part of the dopamine system that is involved in regulation of dopamine receptor‐associated intracellular signaling. The D1R‐interacting protein calcyon and the D2R‐interacting protein neuronal calcium sensor‐1 (NCS‐1) were elevated in the prefrontal cortex of schizophrenia patients,^48^ suggesting that DRIPs might be related to schizophrenia. Research has showed that the expression of DRIPs can affect dopamine receptor trafficking and/or modification of receptor signaling.^49^ Meanwhile, dopaminergic signaling disorders in schizophrenia may be caused by altered expression or function of DRIPs and abnormal interactions between DRIPs and dopamine receptors.^50^ Gm527 gene is conserved in humans, rodents, and amphibians; however, its function is still uncharacterized. Yeast two‐hybrid system identified its binding with D1R.^24^ Here we confirm it is a DRIP of D1R with immunoprecipitation (Figure 1A), and we also observed that its expression was dramatically increased in MK801‐injected mice (Figure 1B), implying its involvement in schizophrenia, consistent with the finding that it might be a schizophrenia‐related gene. To further investigate whether Gm527 can induce schizophrenia‐related phenotypes through its interaction with D1R, we specifically knockout Gm527 in D1R‐positive neurons, and its expression decreased in the DG, but not in the PFC (Figure 1D,E). In addition, FISH indicated that the expression of Gm527 mRNA was ablated in D1R‐positive neurons in the DG of D1:Gm527−/− mice (Figure 1F,G). Consistent with these findings, no hyperlocomotion was observed after Gm527 knockout in D1R‐positive neurons (Figure S1a,b). However, interestingly, spatial working memory and long‐term memories were enhanced (Figure 2), implying that Gm527 might be involved in the cognitive behavior of schizophrenia. In addition, tone‐cued fear conditioning was increased in D1:Gm527−/− mice, but not in AAV‐Gm527‐injected mice, suggesting that other brain regions besides the hippocampus were affected.

The pathological basis of cognitive impairment in schizophrenia is the dysfunction of prefrontal‐striatal‐thalamus‐temporal system. Among them, hippocampal dysfunction plays an important role, because patients with schizophrenia have significant memory and learning deficits that require the participation of neurotransmitters such as dopamine, norepinephrine, acetylcholine, and glutamate. The “dopamine hypothesis” of schizophrenia suggests that dopamine dysfunction in the hippocampus and prefrontal can lead to cognitive impairment, including working memory and episodic memory deficits.^8^, ^9^ The dentate gyrus is the input region of the hippocampus, essential for the representations of sensory information^10^, ^11^ and memory formation.^12^ Adult neurogenesis in the DG is crucial in context discrimination in spatial memory, and its dysregulation is related to schizophrenia.^43^ Ki67 (Figure 3A,B) and BrdU immunostaining (Figure 3C,D) showed increased neural stem cell proliferation in the DG of D1:Gm527−/− mice, suggesting that Gm527 knockout might promote adult neurogenesis in the DG, which might subsequently improve context discrimination in spatial memory.

Dopamine guides the memory encoding^16^ in the DG, which might due to the modulation of D1 receptor in plasticity of glutamatergic neuron synapses from perforant path to DG granular layer.^17^ The increased level of D1R on the membrane facilitates the CREB pathway,^51^ which is required for long‐term synaptic plasticity and memory consolidation.^47^ Activation of the CREB pathway can enhance the expression of GluN2B,^52^ which is important for plasticity at the postsynaptic density.^46^ Our results show that knockout Gm527 in D1R‐positive neurons can increase D1R on the plasma membrane, leading to activation of the CREB pathway in glutamatergic neurons and long‐term plasticity in the DG (Figure 4).

To confirm the importance of the DG in mediating the phenotypes in D1:Gm527−/− mice, we specifically reversed Gm527 expression in D1R‐positive neurons in the DG of these mice (Figure 5A–C), then we observed that D1R reduced on the cell surface and cAMP pathway was inactivated (Figure 7), similarly with another DRIP protein DRIP78.^53^ Subsequently, LTP was inhibited in the perforant path in the DG (Figure 7A,B), and PV‐positive neurons activity was reduced (Figure 6E,F), resulting in long‐term memory and working memory reduced (Figure 5F–K), respectively. The PFC is also critical for cognitive functions, and we also observed that Gm527 was reduced in D1:Gm527−/− mice (Figure 1E), whether Gm527 in the PFC is involved in schizophrenia needs further investigation.

Besides binding with DRIPs, D1R also interacts directly with NMDA receptors,^54^ activation of D1R increases GluN1 and GluN2B cell surface expression,^55^ which might increase the excitability and synaptic plasticity of these neurons. Consistent with these findings, we observed that knockout Gm527 in D1R‐positive neurons increased D1R cell surface expression and GluN2B expression, but not affecting GluN2A (Figure 4C,D); however, after reverse Gm527 expression in D1R‐positive neurons, D1R cell surface expression and GluN2B expression were decreased, whereas GluN2A expression was increased (Figure 7C,D), suggesting that there might be a compensating mechanism to maintain the total NMDA receptors during less D1R surface expression.

Thus, we proposed a “gain‐of‐function” mechanism for Gm527 in schizophrenia cognitive traits via suppression of D1R expression on the plasma membrane; nevertheless, the precise function of Gm527 in schizophrenia still requires extensive further research. The cause of the enhanced C14orf28 expression in schizophrenia patients is unknown, and it must be determined whether polymorphism or mutation of the gene might cause the overexpression. More research is needed to determine the effects of C14orf28 overexpression; however, it is more difficult to manage exogenous overexpression of a gene in animals similar to endogenous overexpression in patients. Therefore, here we employed a conditional knockout strategy to evaluate Gm527 function in schizophrenia‐related phenotypes; however, we did not observe any effects in hyperlocomotion, one of the central phenotypes in the schizophrenia animal model. Gm527's potential involvement in schizophrenia via a D1R‐independent mechanism is a reasonable explanation. Similar to hyperlocomotion, research is needed into additional schizophrenia traits such as social interaction and sensory gating.

## AUTHOR CONTRIBUTIONS

YL conceived the study and participated in the experiment design. JJ performed the experiments, carried out the functional analysis, and drafted the manuscript. HP, RT, and HZ conducted or assisted the study. HZ, BL supervised the study and reviewed the manuscript. All authors contributed to the article and approved the submitted version.

## FUNDING INFORMATION

This study was supported in parts by grants from National Natural Science Foundation of China (82071211, 82071508, 81670930).

## CONFLICT OF INTEREST STATEMENT

The authors declare that they have no competing interests.

## Supporting information


Figure S1

Figure S2

Figure S3

Table S1

Appendix S1

## Data Availability

The datasets during the present study are available from the corresponding author on reasonable request.
