# Supplementary figures and images for: Gm527 deficiency in dentate gyrus improves memory through upregulating dopamine D1 receptor pathway
Source: CNS Neurosci Ther. 2023 May 29;29(11):3290–306. doi: 10.1111/cns.14259 (PMC10580352; doi:10.1111/cns.14259)

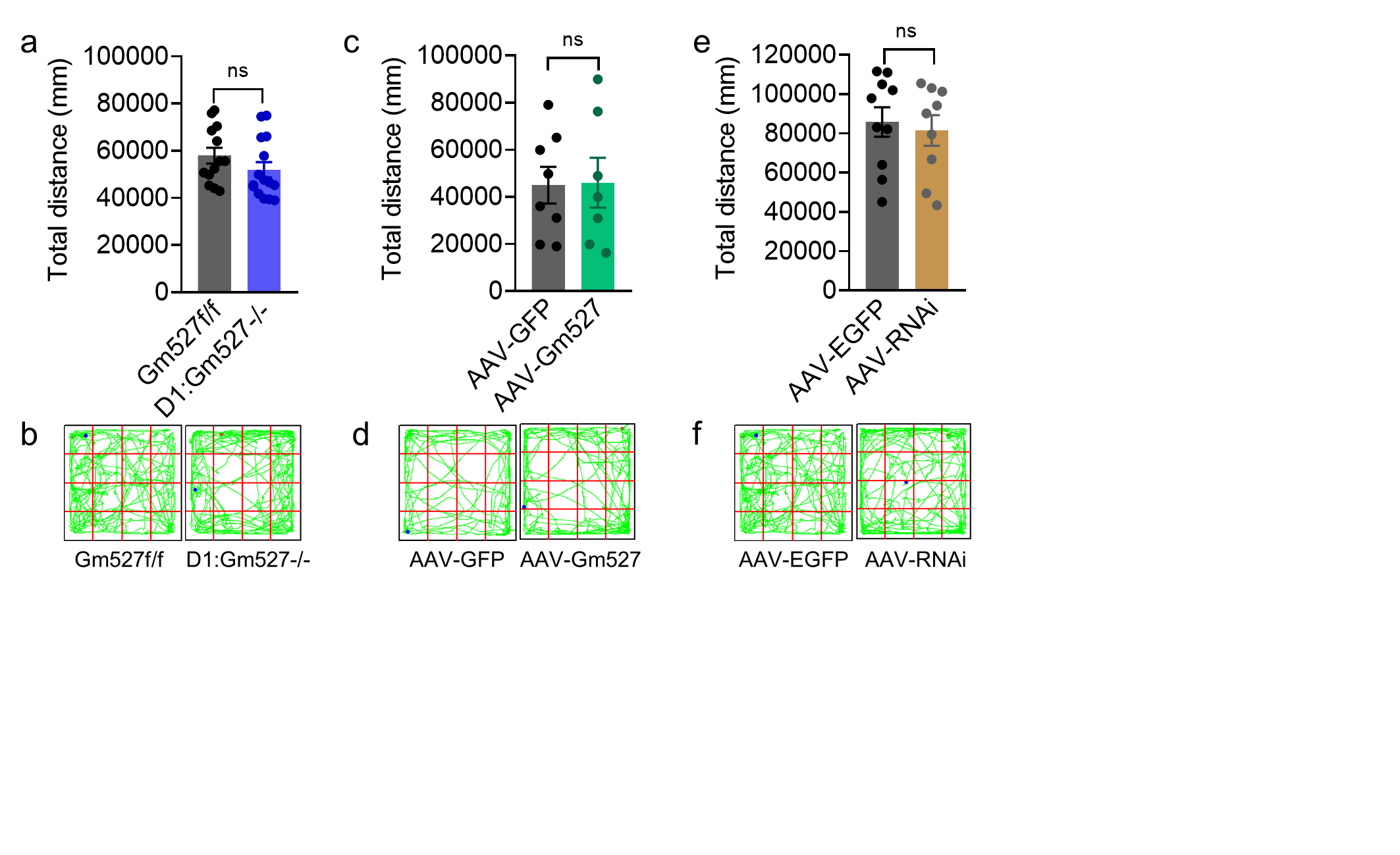

Supplement: Supplementary file 1 — Figure S1 [file CNS-29-3290-s002.tif]

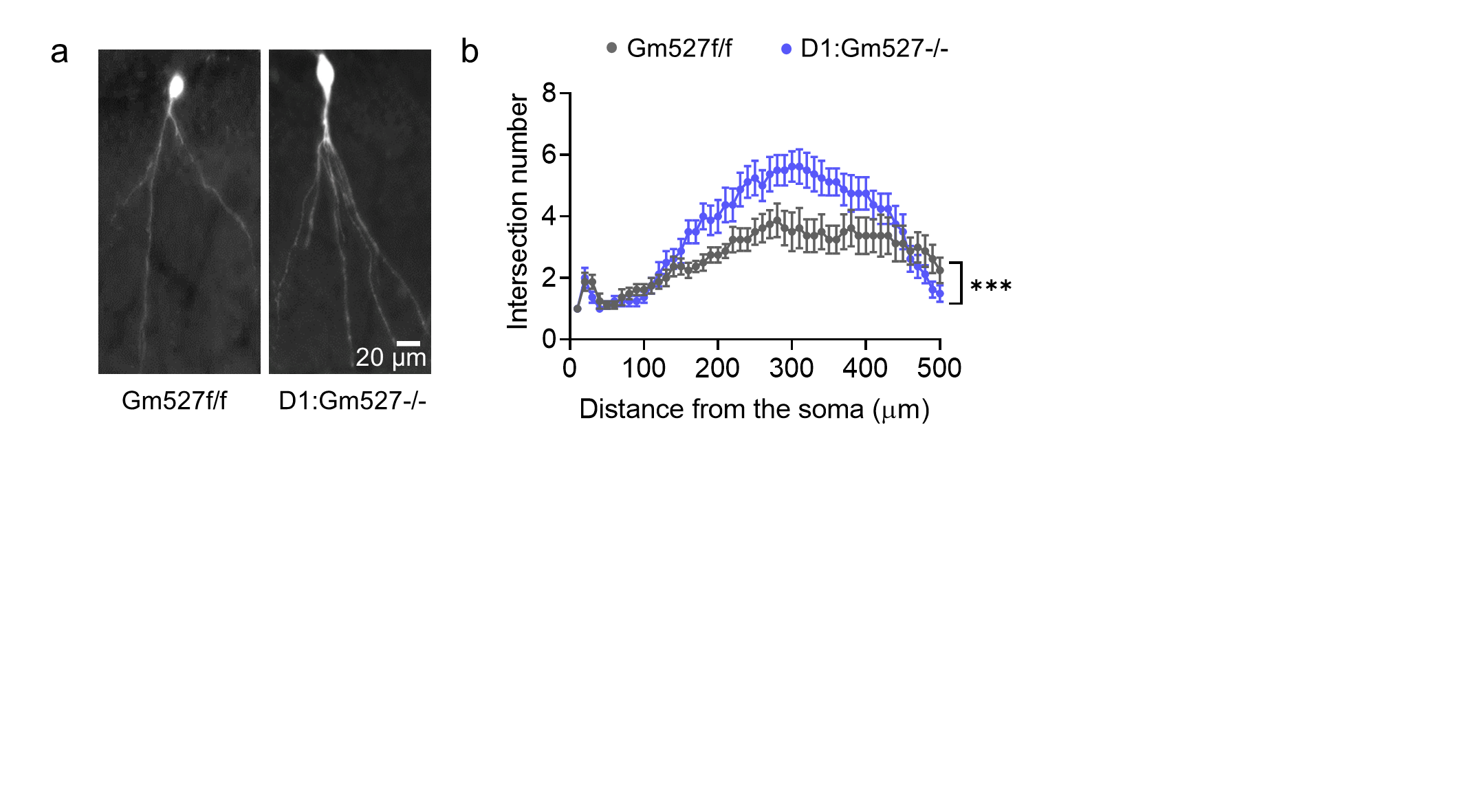

Supplement: Supplementary file 2 — Figure S2 [file CNS-29-3290-s003.tif]

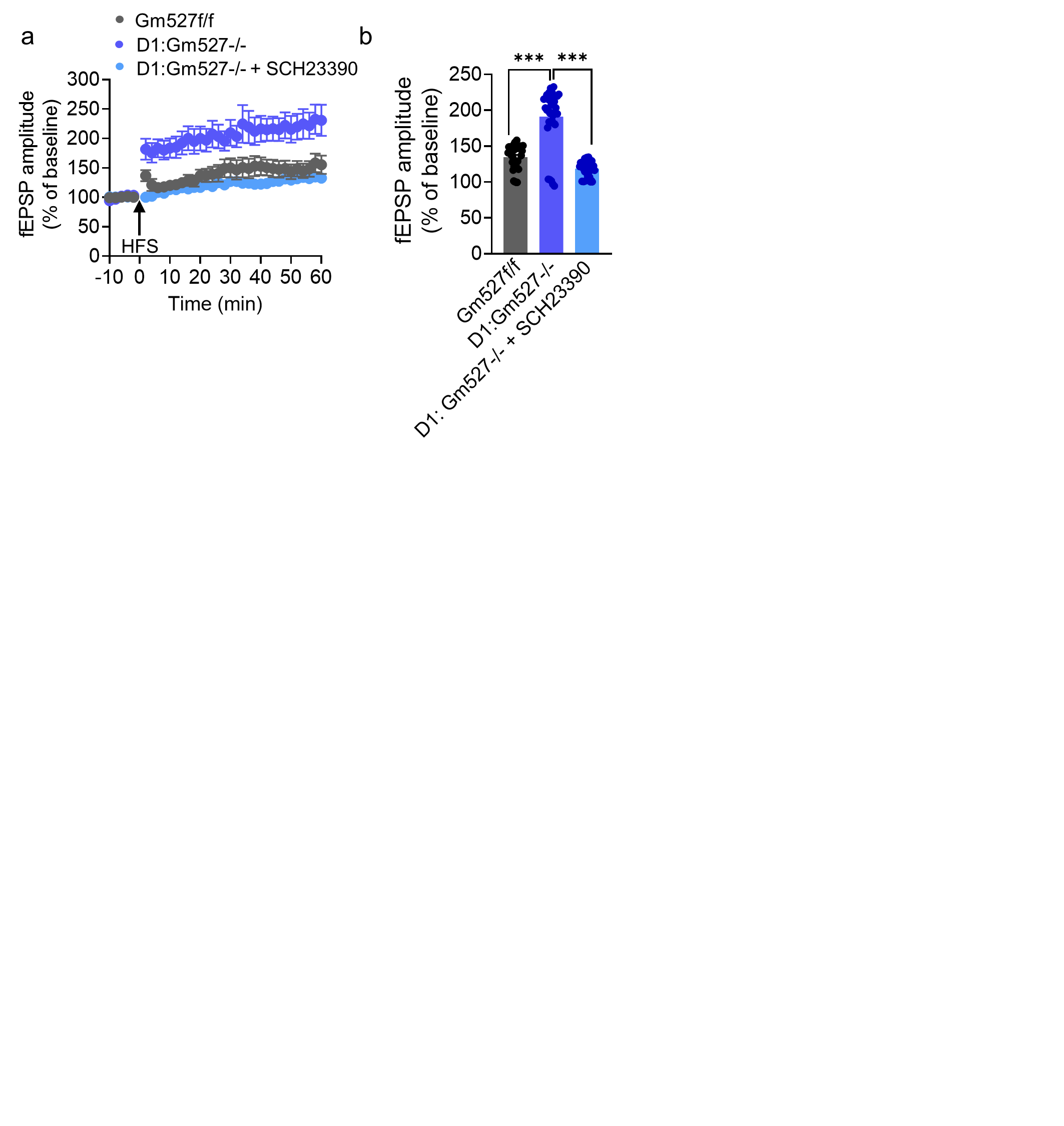

Supplement: Supplementary file 3 — Figure S3 [file CNS-29-3290-s005.tif]
