# Supplementary material for: Gm527 deficiency in dentate gyrus improves memory through upregulating dopamine D1 receptor pathway
Source: CNS Neurosci Ther. 2023 May 29;29(11):3290–306. doi: 10.1111/cns.14259 (PMC10580352; doi:10.1111/cns.14259)
Supplement: Supplementary file 4 — Table S1 [file CNS-29-3290-s001.docx]

**Table S1. Probe sequences (5’→3’) for in situ hybridization.**

**D1R**

| 1 | GCATTCTTTCTTGAGGAGaaGTAGAAGTGTTAGGAGCCAT |
| --- | --- |
| 2 | GCAGGCCAGTCTCATCCATGaaGGCAGCAAACGGGAAGAG |
| 3 | GCATTCTTTCTTGAGGAGaaATTCCCTAAGAGAGTGGACA |
| 4 | ACAGCGGCACAGACAAGGGTaaGGCAGCAAACGGGAAGAG |
| 5 | GCATTCTTTCTTGAGGAGaaCAGCTAAAGAGATGACAAAG |
| 6 | AGCCACCAAGAGATCTGACAaaGGCAGCAAACGGGAAGAG |
| 7 | GCATTCTTTCTTGAGGAGaaGGATGGATGCCGTGGAGCAC |
| 8 | GCTGATCACACAGAGGTTAAaaGGCAGCAAACGGGAAGAG |
| 9 | GCATTCTTTCTTGAGGAGaaATGAAGGCTGCCTTCGGAGT |
| 10 | TCCATGCTACGCTAATCAGGaaGGCAGCAAACGGGAAGAG |
| 11 | GCATTCTTTCTTGAGGAGaaCACTGGAATGAAGGATATGA |
| 12 | GCCTTGTGCCAGCTTAGCTGaaGGCAGCAAACGGGAAGAG |
| 13 | GCATTCTTTCTTGAGGAGaaAAATTGCCATCCAAGGGCCA |
| 14 | CGGCATCTTCCAGGGAAGTAaaGGCAGCAAACGGGAAGAG |
| 15 | GCATTCTTTCTTGAGGAGaaACGATCATAATGGCTACGGG |
| 16 | TGTAGATACTGGTGTAAGTGaaGGCAGCAAACGGGAAGAG |
| 17 | GCATTCTTTCTTGAGGAGaaGCCGGATTTGCTTCTGGGCA |
| 18 | CCTCTCCAAAGCTGAGATGCaaGGCAGCAAACGGGAAGAG |
| 19 | GCATTCTTTCTTGAGGAGaaTTCCATTACCTGTGGTGGTC |
| 20 | TTGAGAGCATTCGACAGGGTaaGGCAGCAAACGGGAAGAG |
| 21 | GCATTCTTTCTTGAGGAGaaTCCCTCTTAAAGGACATCTT |
| 22 | GTGTCTTCAGGACTTTAGTCaaGGCAGCAAACGGGAAGAG |
| 23 | GCATTCTTTCTTGAGGAGaaGAATCAATGCAGAATGGCTG |
| 24 | CAAATACATCGAAGGTGATGaaGGCAGCAAACGGGAAGAG |
| 25 | GCATTCTTTCTTGAGGAGaaCCTCCTCCCTCTTCAGGTCC |
| 26 | TGGCTTAGGTATGCCACCGGaaGGCAGCAAACGGGAAGAG |
| 27 | GCATTCTTTCTTGAGGAGaaTCTAGAGAGACATCGGTGTC |
| 28 | GGGTAACGGGTTGGATCTTTaaGGCAGCAAACGGGAAGAG |

**Gm527**

| 1 | CACATTTACAGACCTCAAtaCGGCCAGCTTTGATAGTAAA |
| --- | --- |
| 2 | GTGTACAAGATACTGCTTTGaaCCTACCTCCAACTCTCAC |
| 3 | CACATTTACAGACCTCAAtaAAGCAGCTTCACAGTTGTAA |
| 4 | TCCATCTTGTCCCAAATCCAaaCCTACCTCCAACTCTCAC |
| 5 | CACATTTACAGACCTCAAtaTAGGGCAGTGCCAGTTAGAG |
| 6 | CTGATCTTCCTGCACTCGGGaaCCTACCTCCAACTCTCAC |
| 7 | CACATTTACAGACCTCAAtaTCCATGTGGAATTTCTTGGG |
| 8 | ACAGCCAGTCGTATCAAGTTaaCCTACCTCCAACTCTCAC |
| 9 | CACATTTACAGACCTCAAtaGTCTTCAGGTTTCCAATGCT |
| 10 | TAATAGGGTACATATGCCAGaaCCTACCTCCAACTCTCAC |
| 11 | CACATTTACAGACCTCAAtaTTGTTAAATAAAGTGGCGCC |
| 12 | CAGAATAATGATGCTCCTCTaaCCTACCTCCAACTCTCAC |
