# Supplementary material for: Gm527 deficiency in dentate gyrus improves memory through upregulating dopamine D1 receptor pathway
Source: CNS Neurosci Ther. 2023 May 29;29(11):3290–306. doi: 10.1111/cns.14259 (PMC10580352; doi:10.1111/cns.14259)

Full unedited gel/blot for Figure 1a

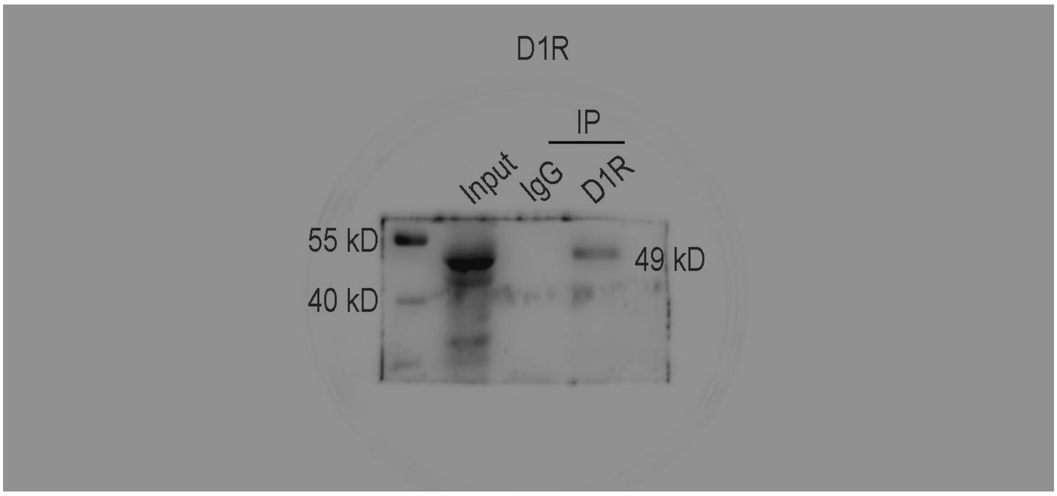

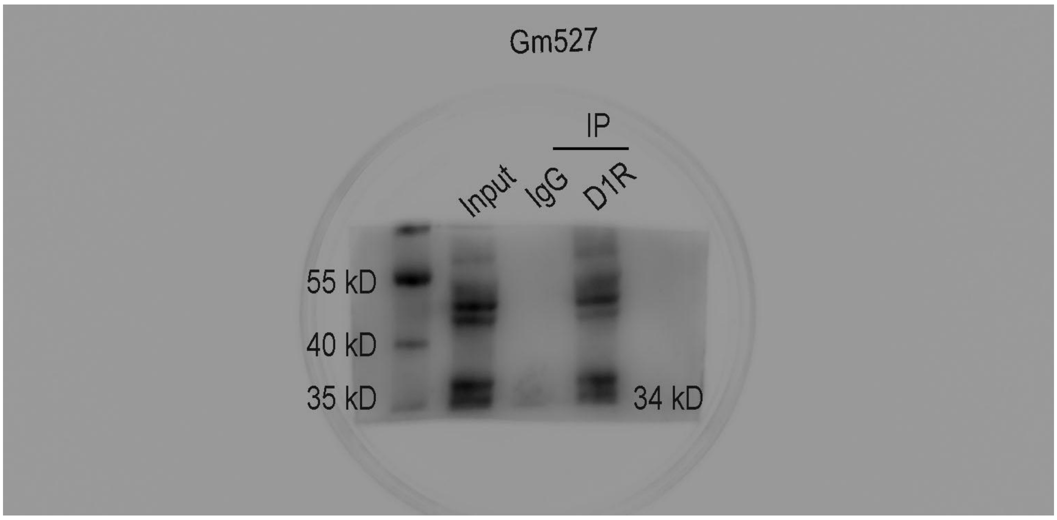

Full unedited gel/blot for Figure 1b

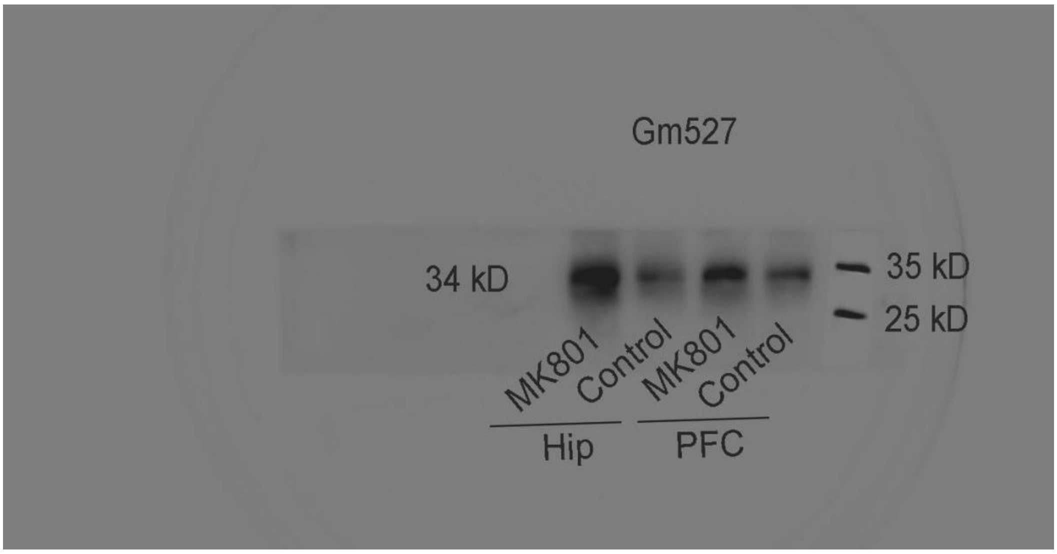

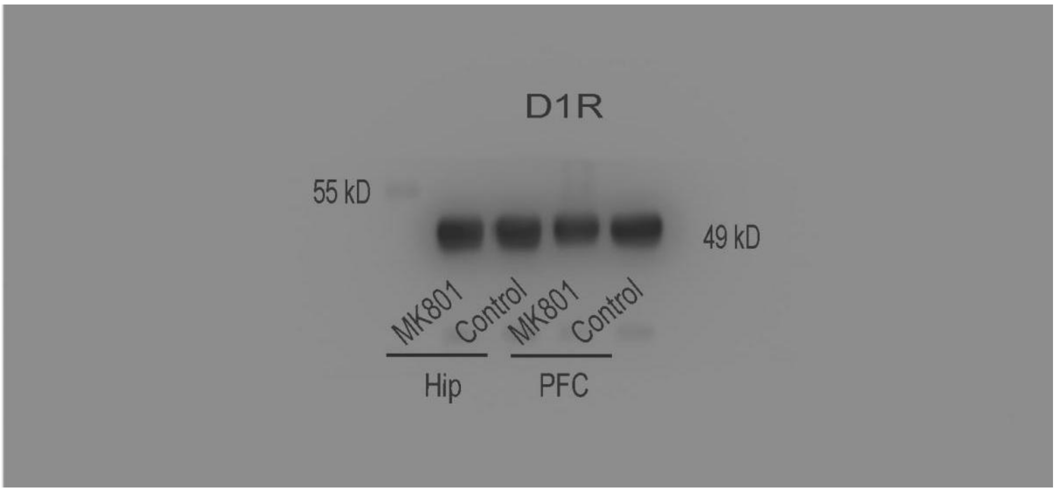

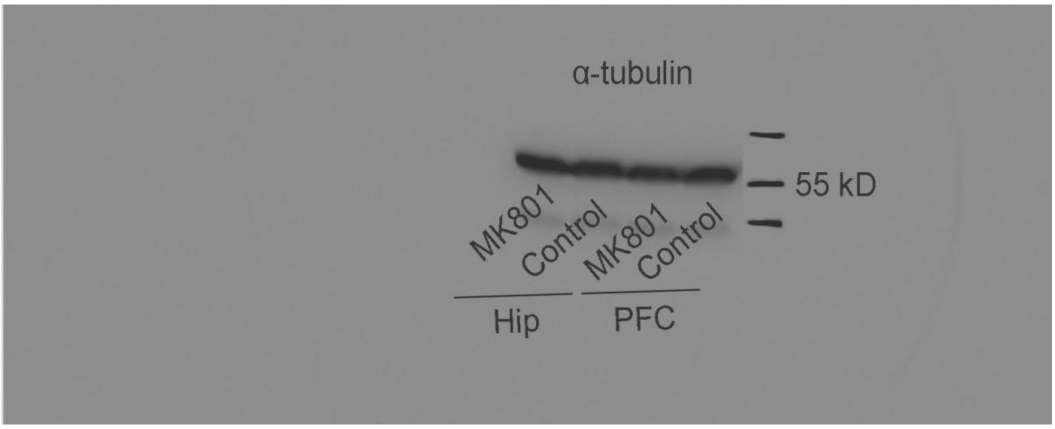

Full unedited gel/blot for Figure 1e

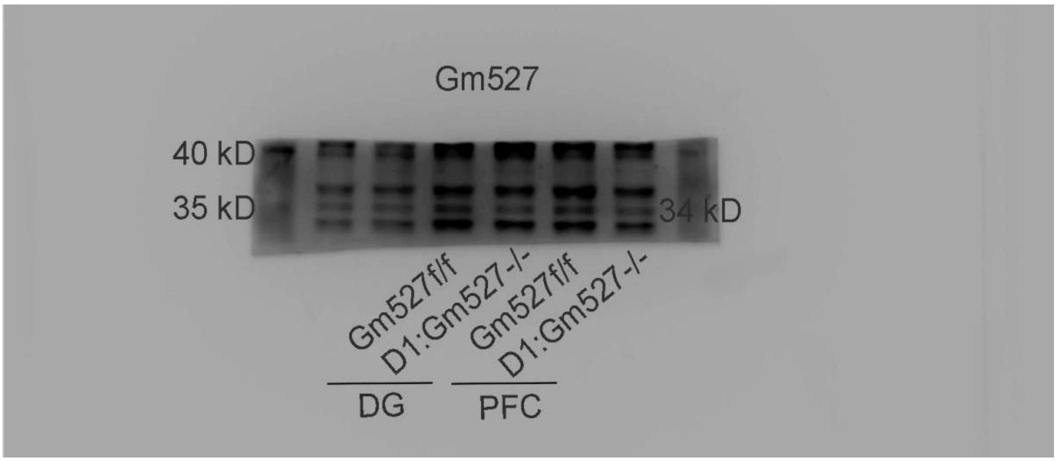

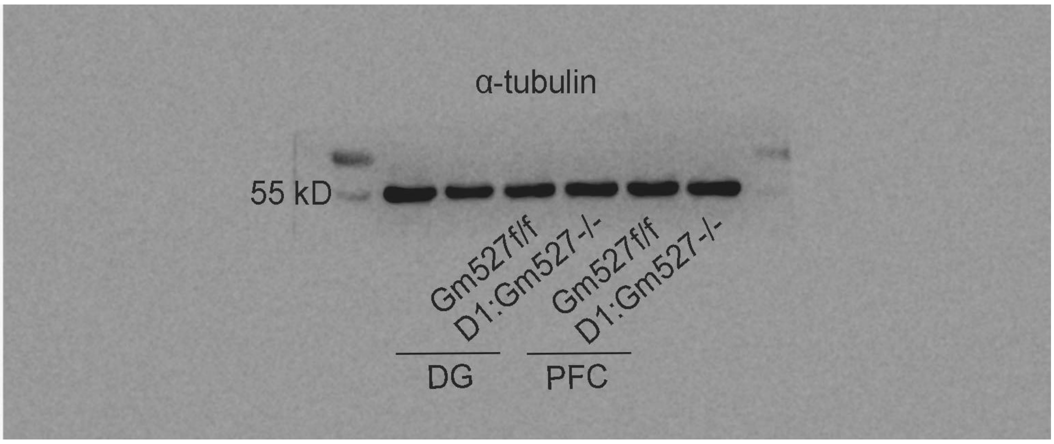

Full unedited gel/blot for Figure 4c

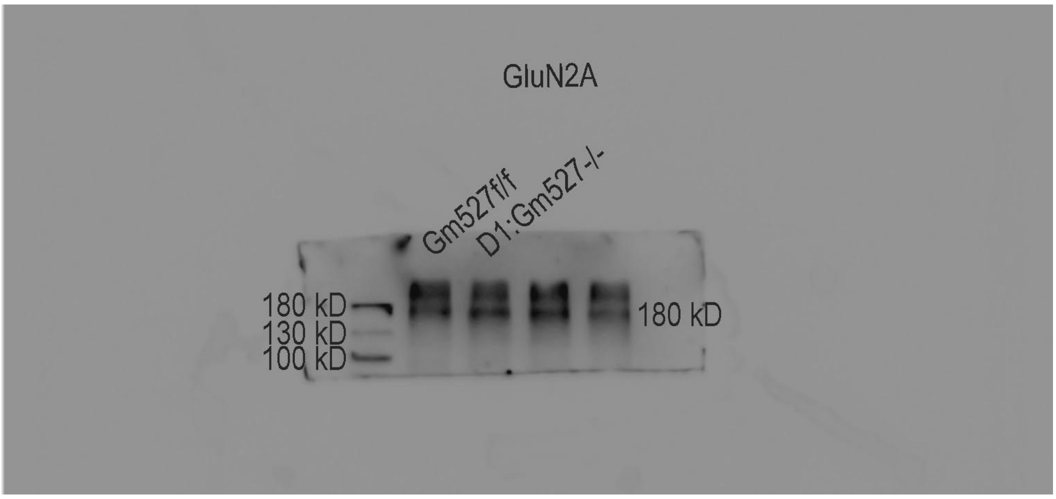

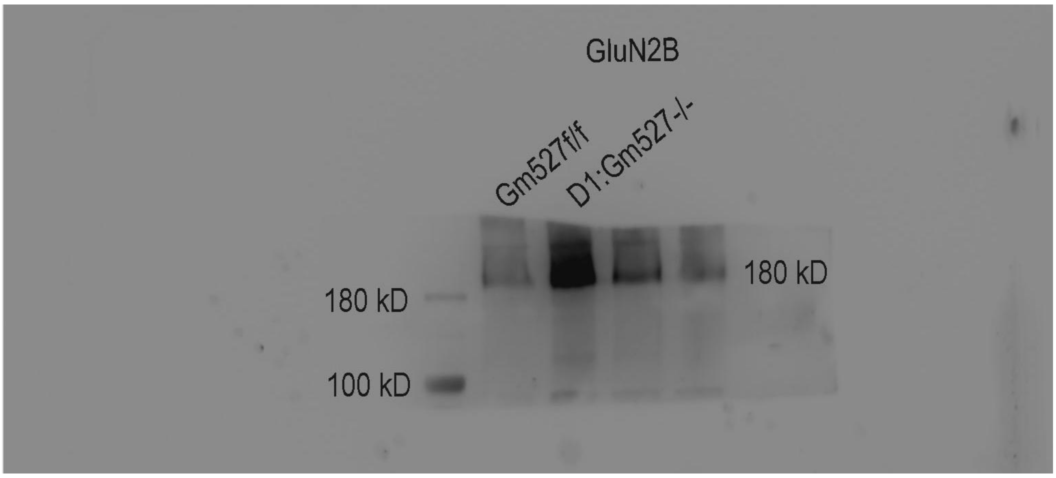

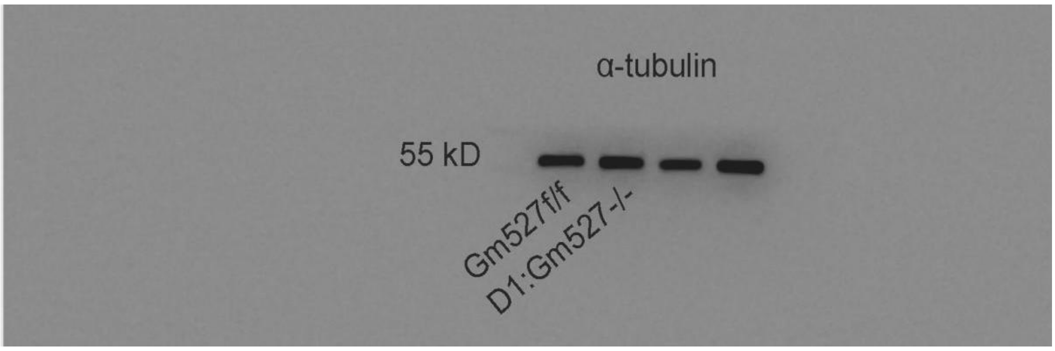

Full unedited gel/blot for Figure 4e

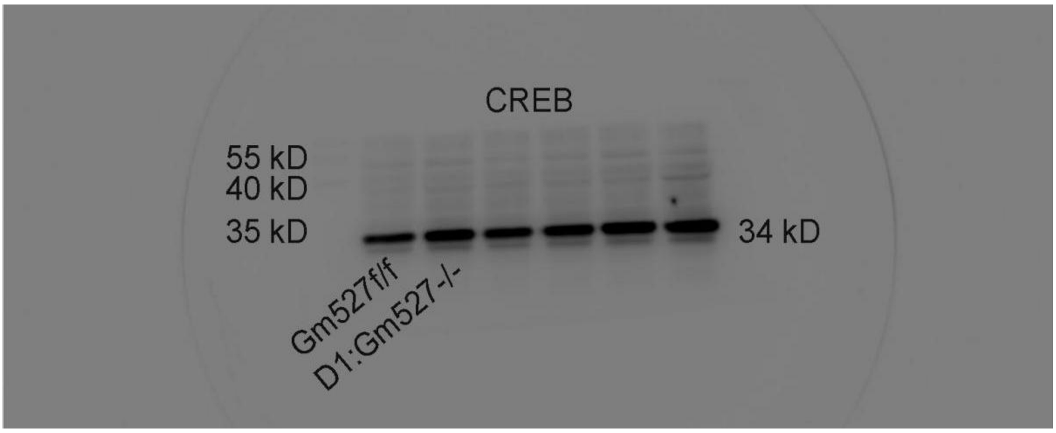

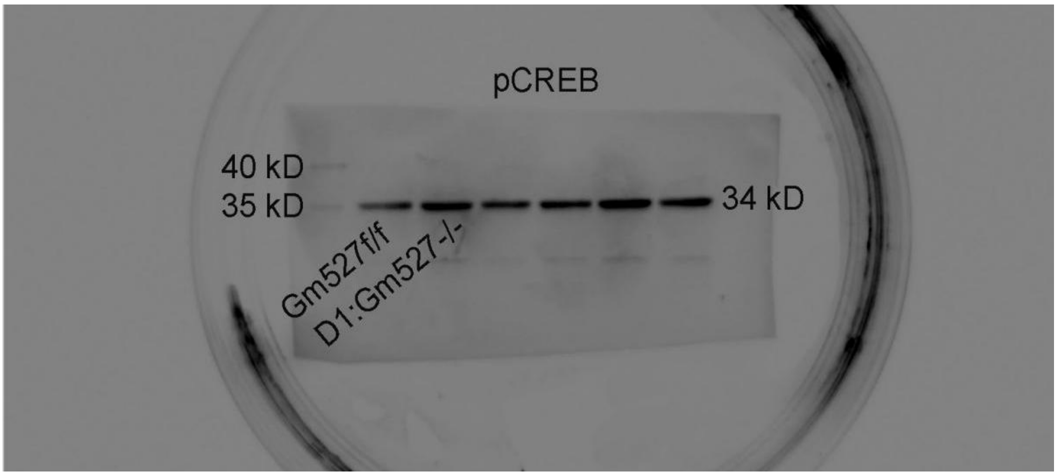

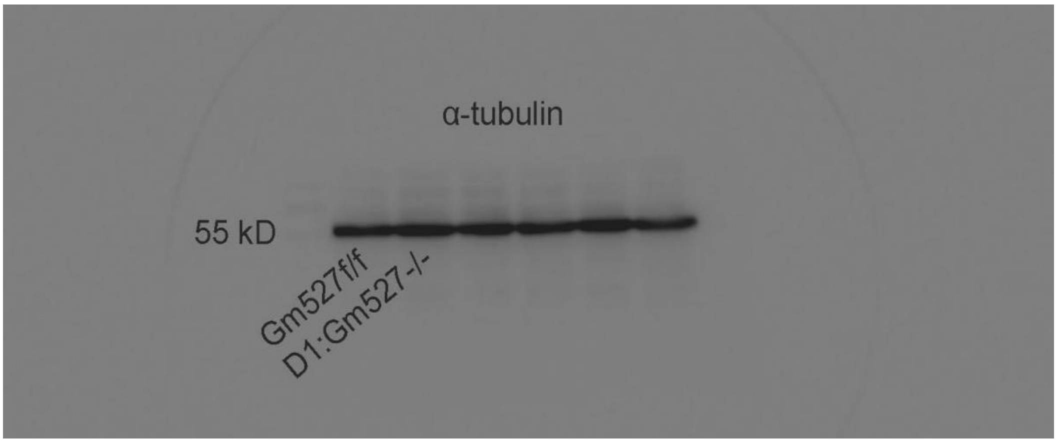

Full unedited gel/blot for Figure 4g

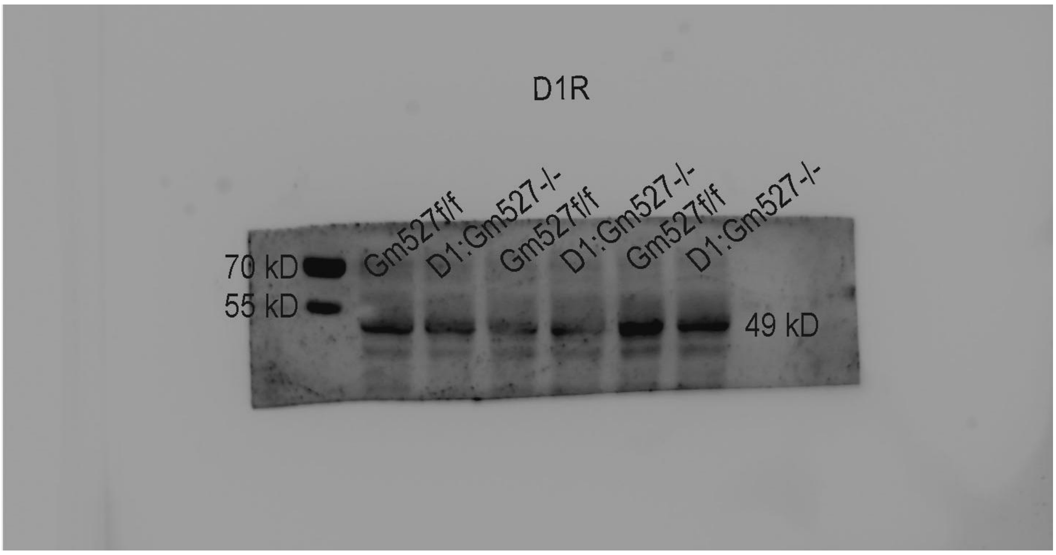

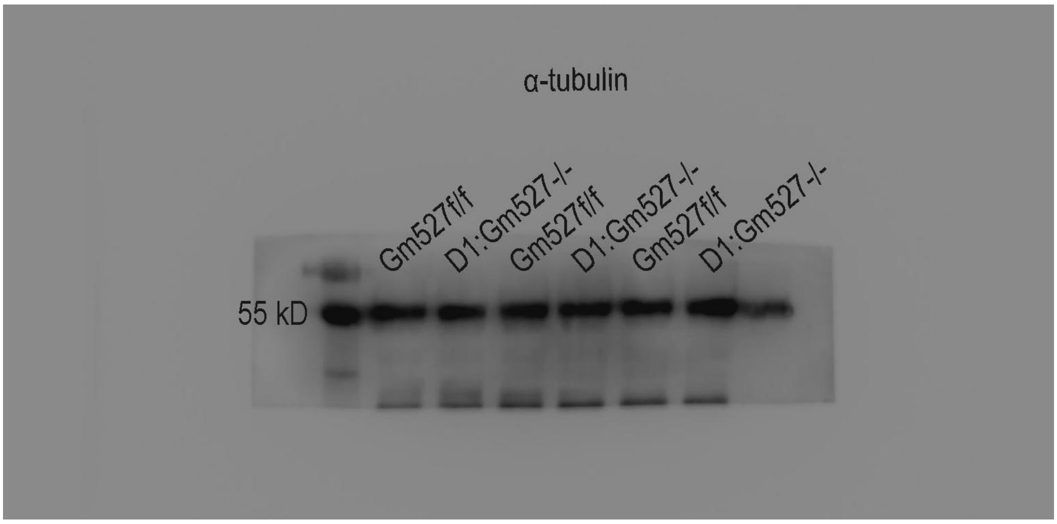

Full unedited gel/blot for Figure 4i

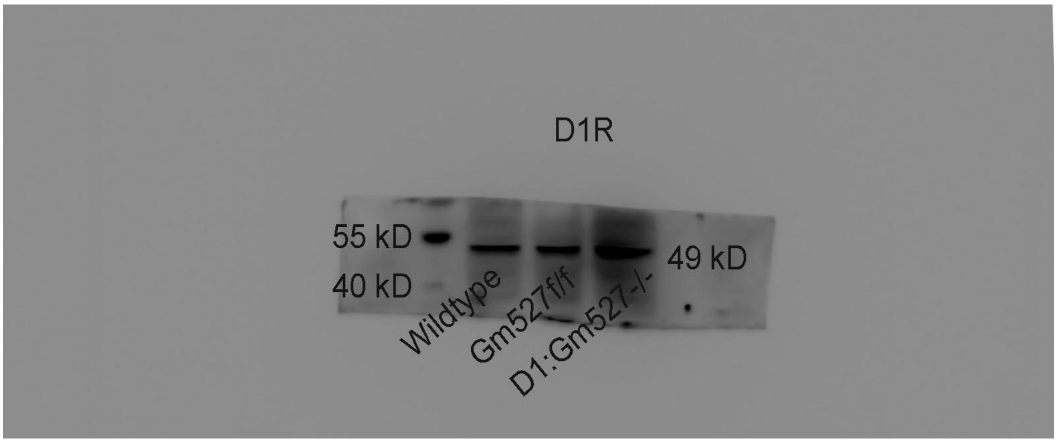

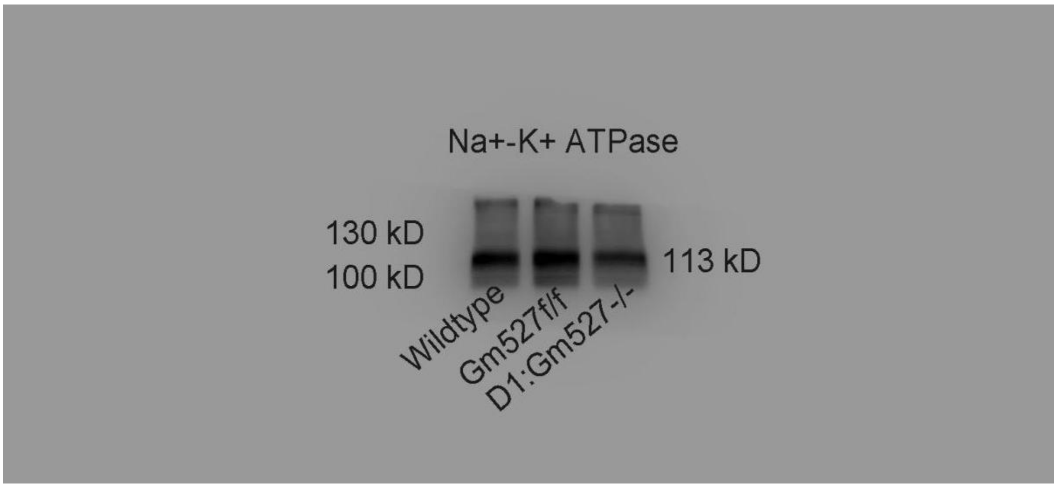

Full unedited gel/blot for Figure 5c

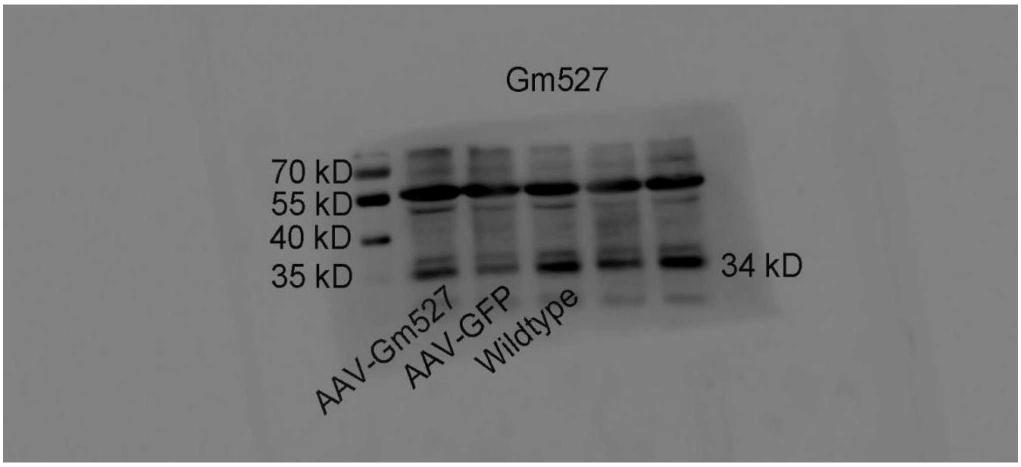

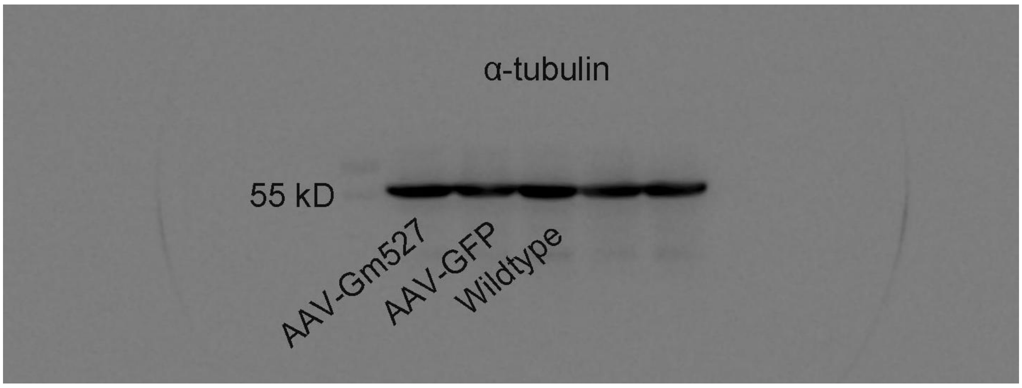

Full unedited gel/blot for Figure 7c

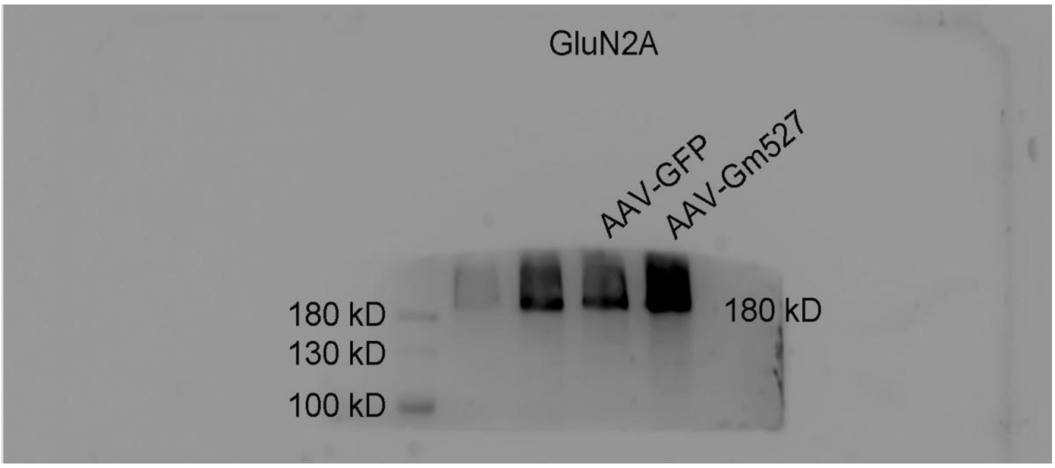

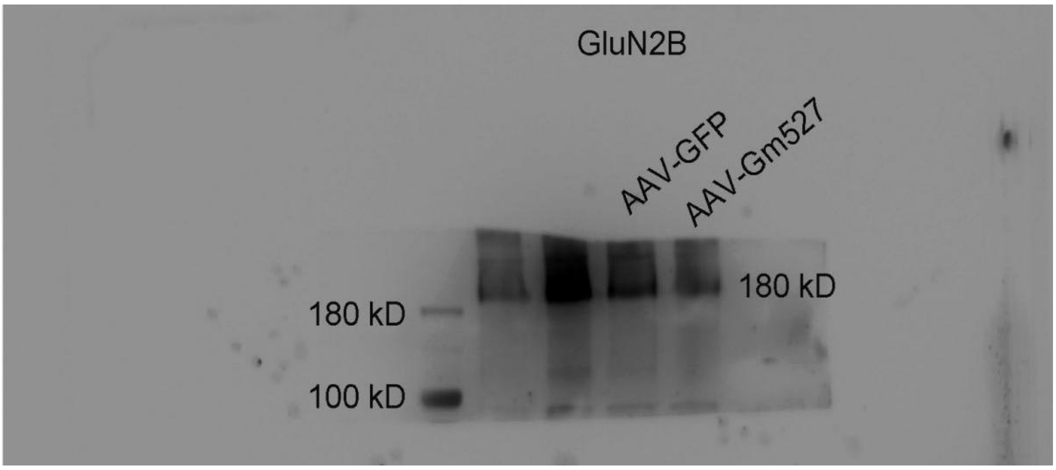

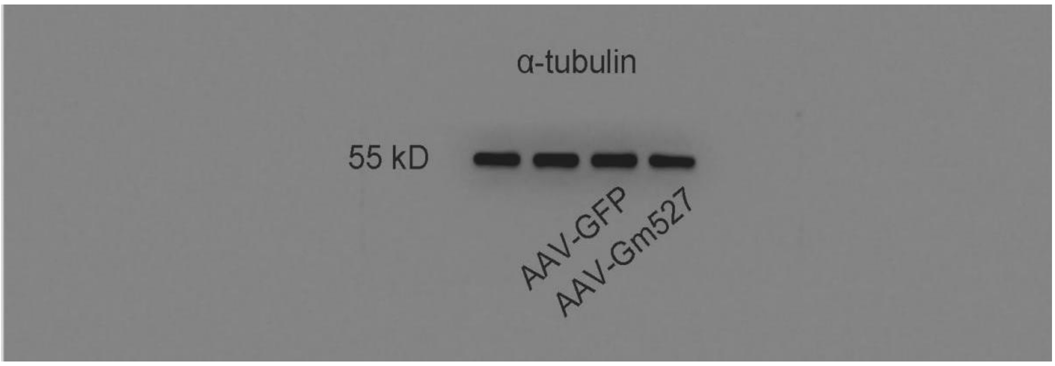

Full unedited gel/blot for Figure 7e

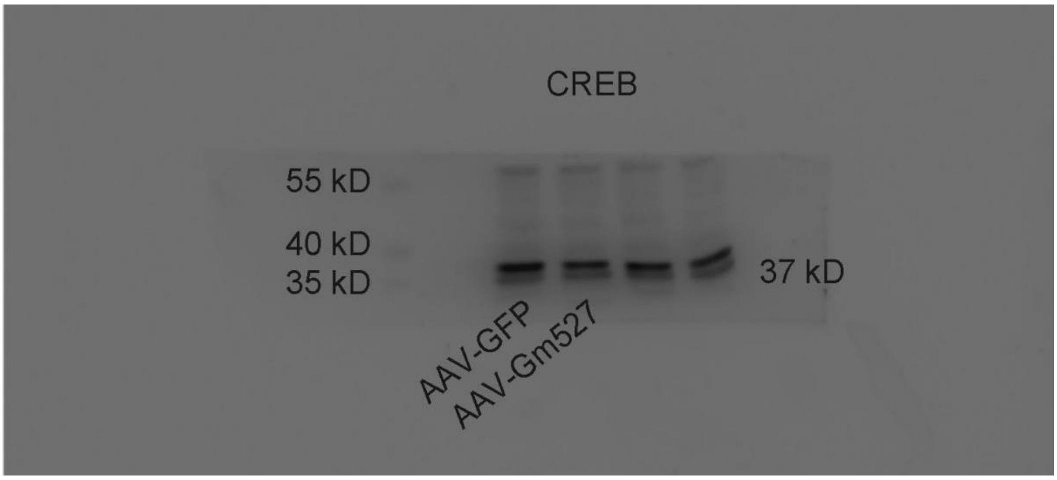

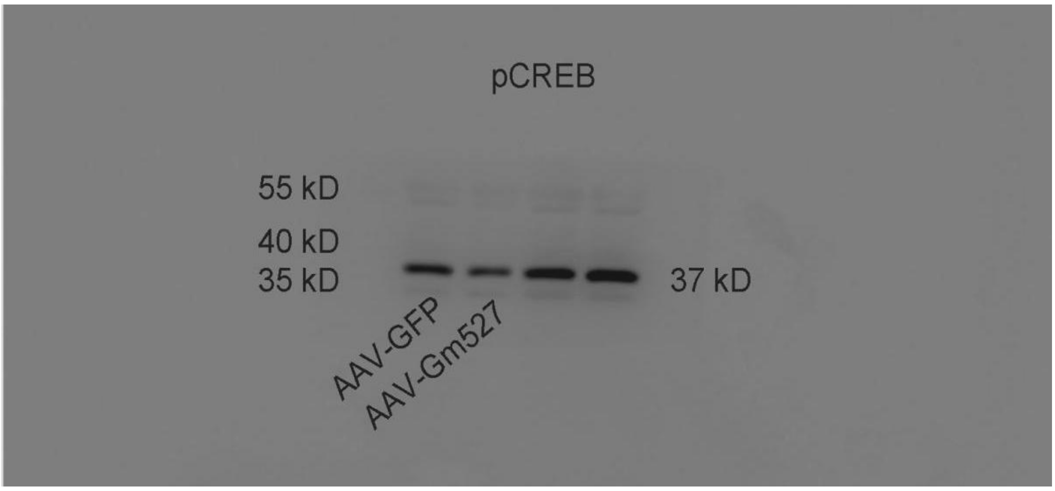

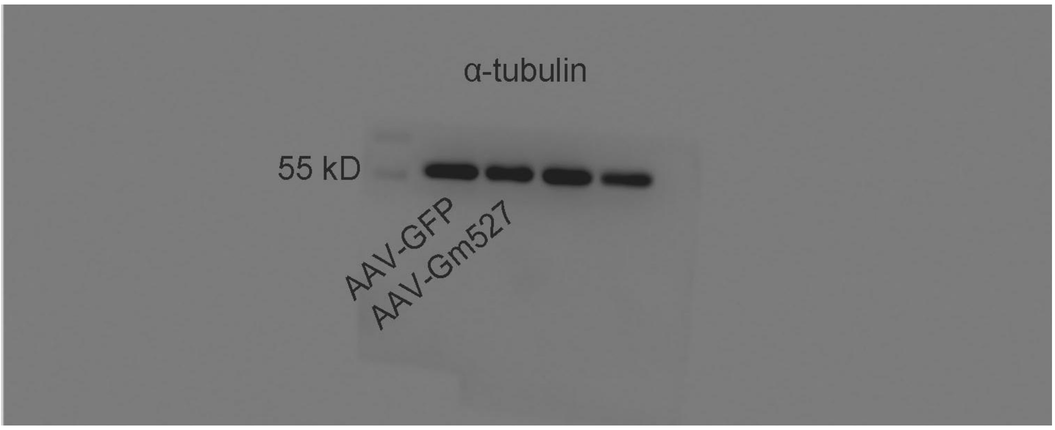

Full unedited gel/blot for Figure 7g

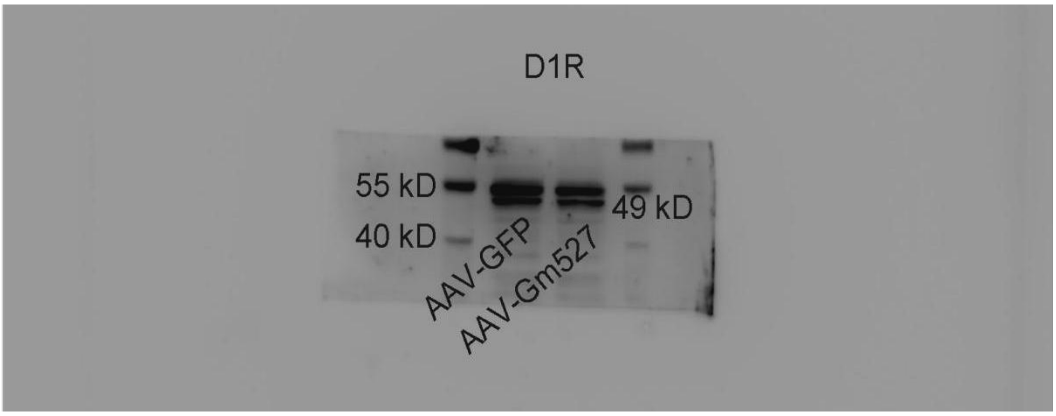

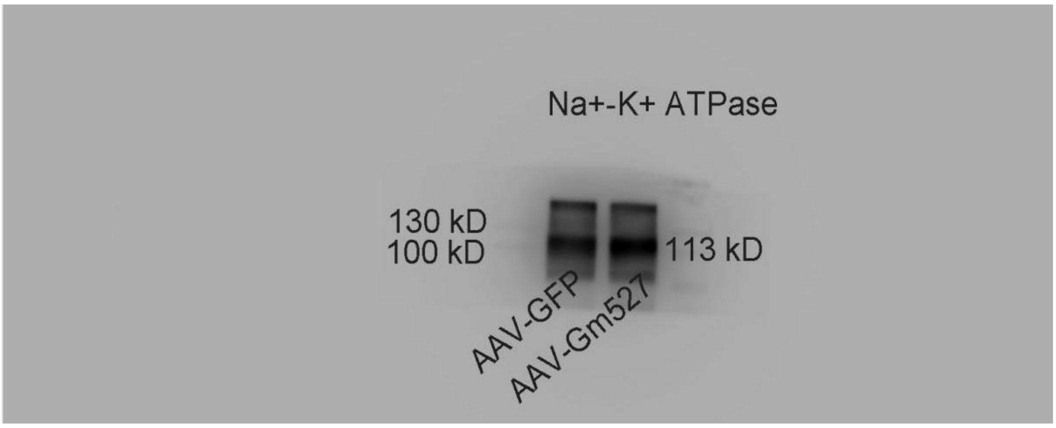

Full unedited gel/blot for Figure 8c

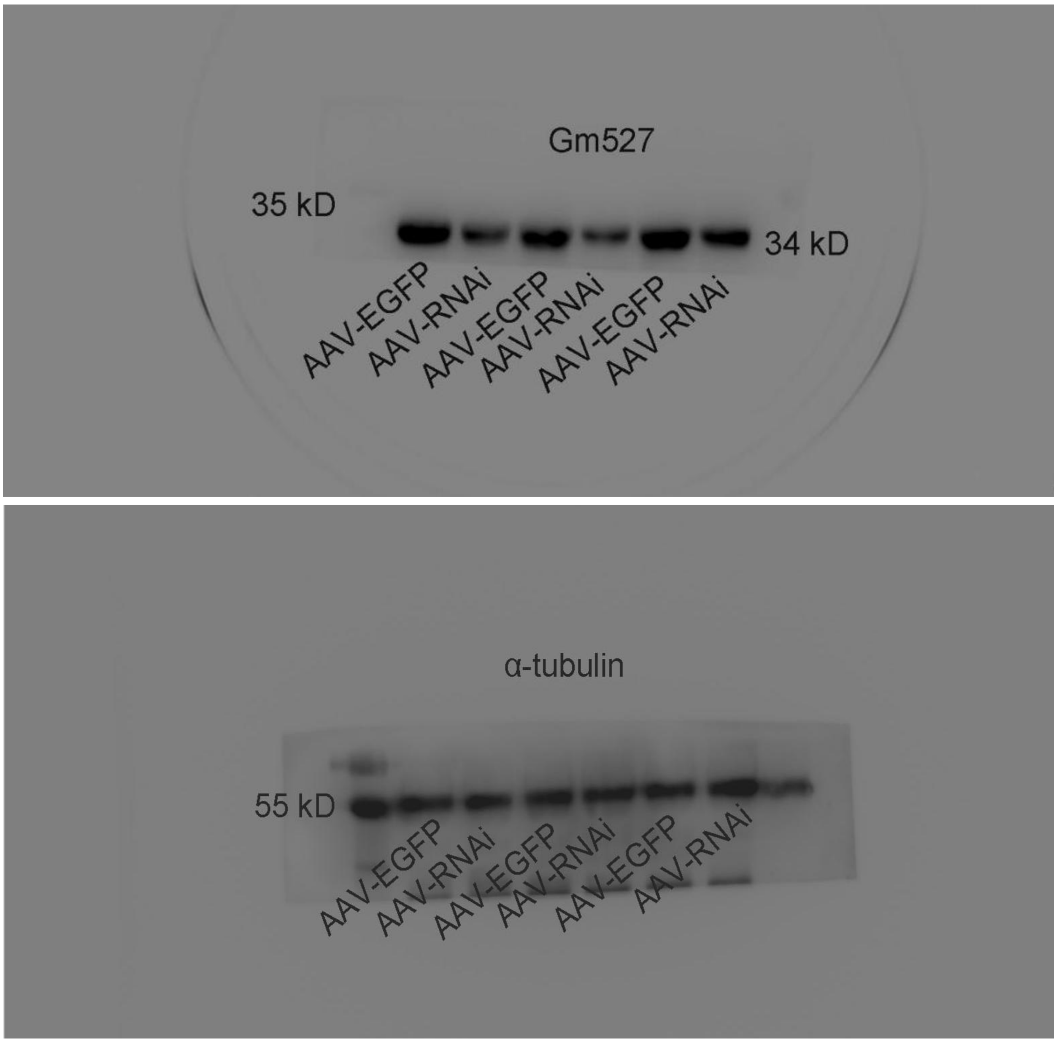

Supplement: Supplementary file 5 — Appendix S1 [file CNS-29-3290-s004.pdf]
